# Self-assembled porous polymer films for improved oxygen sensing

**DOI:** 10.1016/j.snb.2022.132794

**Published:** 2023-01-01

**Authors:** Nikolaos Salaris, Paul Haigh, Ioannis Papakonstantinou, Manish K. Tiwari

**Affiliations:** a Nanoengineered Systems Laboratory, UCL Mechanical Engineering, University College London, London WC1E 7JE, United Kingdom; b Wellcome/EPSRC, Centre for Interventional and Surgical Sciences (WEISS), University College London, London W1W 7TS, United Kingdom; c School of Engineering, Newcastle University, Newcastle, NE1 7RU, United Kingdom; d Photonic Innovations Lab, Department of Electronic & Electrical Engineering, University College London, London WC1E 7JE, United Kingdom

**Keywords:** Porous polymer films, Phase inversion and breath figure method, Phosphorescence based oxygen sensing, Improvement of sensing properties

## Abstract

Absolute oxygen sensors based on quenching of phosphorescence have been the subject of numerous studies for the monitoring of biological environments. Here, we used simple fabrication techniques with readily available polymers to obtain high performance phosphorescent films. Specifically, evaporation-based phase separation and the breath figure technique were used to induce porosity. The pore sizes ranged from ∼37 nm to ∼141 μm while the maximum average porosity achieved was ∼74%. The oxygen sensing properties were evaluated via a standarised calibration procedure with an optoelectronic setup in both transmission and reflection based configurations. When comparing non-porous and porous films, the highest improvements achieved were a factor of ∼7.9 in dynamic range and ∼7.3 in maximum sensitivity, followed by an improved linearity with a half-sensitivity point at 43% O${}_{2}$ V/V. Also, the recovery time was reduced by an order of magnitude in the high porosity film and all samples prepared were not affected by variations in the humidity of the surrounding environment. Despite the use of common polymers, the fabrication techniques employed led to the significant enhancement of oxygen sensing properties and elucidated the relation between porous film morphologies and sensing performance.

## Highlights

• Four types of porous polymer films were fabricated.
• Porosity was induced via phase inversion and the breath figure technique.
• Significant improvement of oxygen sensing properties was achieved.
• Film morphology was linked to sensing properties.

## 1 Introduction

Oxygen sensing plays a vital role in the understanding of aerobic processes and has a variety of applications such as in healthcare monitoring [1], cancer research [2], [3], environmental studies [4] and food packaging [5], [6], among others. Recent studies emphasise on optical methods of detection due to their advantages over the gold standard, electrochemical measurement system; the Clark Electrode [7], [8].

The most common technique involves an optoelectronic setup designed to monitor the quenching of the phosphorescence emitted from oxygen sensitive dyes [9]. This is achieved by using an optical source and a receiver alongside the luminescent material, which is placed in contact with the environment under study in order to quantify the concentration of oxygen [10]. These luminescent indicators exhibit quenching of their phosphorescence, i.e long lived luminescence from excited triplet states, through non-radiative deactivation [11], [12], [13]. The phenomenon originates from molecular interactions and is mainly attributed to collisional processes between the phosphorescent dye and oxygen molecules, which act as the quencher [14]. The two main types of measurements rely on intensity of emission and lifetime of the emission decay. Both can be described in the ideal case by the Stern–Volmer equation (Eq. (1)) [12], [15], [16].

(1) $$\frac{I_{o}}{I},\frac{\tau_{o}}{\tau }=1+K_{SV}\left[O_{2}\right]$$

where $I_{o}$, I and are the emitted luminescence intensity and $\tau_{o}$, τ the decay lifetimes in the absence and presence of the quencher, respectively. $K_{SV}$ is the Stern–Volmer constant and $\left[O_{2}\right]$ is the quencher concentration. It should be noted that the partial pressure is linearly correlated with the concentration due to Henry’s law [17], [18].

A multitude of studies have focused on improving the characteristics of phosphorescent dyes in terms of quantum yield, photostability and wavelength of maximum absorption and emission depending on the intended application [19], [20], [21], [22]. Metalloporphyrins are among the most commonly used dyes due to their long lived decays and strong luminescence. Specifically, platinum octaethylporphyrin (PtOEP) has been extensively studied due its favourable characteristics of high quantum yield, long decay lifetime and emission/absorption peak within the visible range [23], [24].

In the majority of sensing applications, the phosphorescent dye requires immobilisation onto a polymer for mechanical stability [25], [26]. The encapsulation of the dye onto the polymer affects most of the oxygen sensing properties of interest. The main characteristics investigated to evaluate the performance of a sensor are: the response/recovery time, defined as the time period necessary for the stabilisation of measurements from one level to another, the sensitivity, usually defined as the ratio of the observed variables (lifetime or intensity) in the absence and presence of the analyte, and the range of detectable oxygen levels [25], [27], [28]. Importantly, at high oxygen levels, the linear relationship described in Eq. (1) ceases to remain valid and the Stern–Volmer plot exhibits a saturation when the indicators are placed in a microheterogeneous environment [29], [30], [31], which is inherently true for polymer support matrices, under inadequate luminescence, due to use of low sensitivity receiver devices and/or dye aggregation [32].

It is common to empirically fit the resulting curves with a multi site model (Eq. (2)), in most cases the two site model, based on the assumption that different binding sites exhibit different solubilities. This has also been shown to be equivalent to the non-linear solubility model [33] [27], [34]:

(2) $$\frac{I_{0}}{I},\frac{\tau_{o}}{\tau }={\left(\overset{}{\underset{j=1}{\sum }}\frac{f_{j}}{1+K_{SV}^{j}\left[O_{2}\right]}\right)}^{-1}$$

where $f_{j}$ is the fraction of each site j and their sum is equal to 1 (for two site model j=1,2). These fit parameters provide a metric for the sensitivity and the range of oxygen sensing measurements.

Numerous studies have focused on achieving favourable sensing characteristics via the correct choice and manipulation of the polymer host [10]. Most important properties of the host polymer are: the permeability to oxygen, film thickness and the solubility in solvents for the phosphorescent dyes [35], [36], [37], [38]. A reduction in the response time and a simultaneous increase in sensitivity has been achieved by using high surface area and high accessibility to gas molecules polymer host supports [39]. Microporosity has been presented as a solution that combines the aforementioned qualities along with the simultaneous increase of scattered light inside the film, which leads to a higher luminescence [39], [40].

In one study, porosity was introduced to the polymer/dye film via ternary phase separation of polyethylene glycol (PEG)/polystyrene (PS)/chloroform solutions [41], [42]. This technique relies on solution-based film fabrication and resulted in a ∼4 fold increase in photoluminescence due to increased scattering from the voids formed. It also led to an increase of up to 72% in sensitivity [39]. In the same study, it was shown that surface porosity played a pivotal role in increasing the sensitivity, possibly due to higher accessibility of oxygen molecules and shortening of the outer layer diffusion barrier. The resulting phosphorescent film was affected by relative humidity but this was overcome with the substitution of PEG for ethyl cellulose [43].

Similarly, the breath figure method is a self-assembly technique that has been widely applied for the formation of porous films. This relies on water droplet condensation on the surface of polymer/solvent blends that are left to evaporate in humid environments [44]. Briefly, the physical mechanism encompasses: (i) evaporation of the polymer solution; (ii) nucleation and condensation of water droplets on the surface of the solution as the temperature drops below the dew point; (iii) magnification and organisation of water droplets in patterns due to Marangoni and capillary forces; (iv) evaporation of the solvents and the water droplets; and (v) solidification of the polymer [45], [46]. The resulting films exhibit micro- and nano-porosity and with the appropriate conditions can form highly ordered structures [47].

Another common approach to increase the sensing capabilities of oxygen indicators is the doping of the polymer films with high dielectric constant particles. Longer optical path lengths lead to an increase in excitation light scattering, which in turn increases light absorption in the dye particles. Other solutions emphasised on nanoparticle formation using amphiphilic acrylamide-based polymers resulting in additional superhydrophobic properties [48]. The construction of microstructured PtTFPP/PDMS pillar arrays using photolithography has also been proposed as a cost effective method of monitoring highly anoxic environments [49].

The implementation of robust and economical fabrication techniques while optimising the sensing characteristics is vital for newly emerging fields such as transcutaneous oxygen sensors for wearable applications [1], [50], [51], [52] and food packaging [6], [53], [54], [55], [56]. In this direction, it is pivotal that reliable and low cost sensors are developed for wide-scale commercial availability. Key features should include mechanical stability, high sensitivity, suitable detectable range and photostability combined with cost efficient materials and fabrication processes. Notwithstanding the above reports achieving these features, simple strategies to design porous polymer films and their impact on increasing sensing characteristics are still lacking.

In this study, porosity was induced in readily available polymers with self-assembly techniques to enhance the oxygen sensing properties of phosphorescent films. This was performed with the drying of solution based ternary systems (solvent/non-solvent/polymer) and the breath figure method by relying on simple drop casting to create polymer films. The morphology of the films was characterised based on image processing techniques and was related to the sensing performance with the use of an optoelectronic setup and a standarised calibration procedure. It is shown that despite the use of simple fabrication procedures and common polymers, a wide range of porosities was achieved leading to significant improvements in oxygen sensing capabilities.

## 2 Experimental procedures

The phosphorescent dye PtOEP was embedded in both porous and dense (i.e. non-porous) films using two different host polymers. Porosity was introduced via two separate evaporation-based techniques and a comparison was performed to link the nature of porosities to the oxygen sensing properties. An image analysis was performed for the characterisation of the film morphology and calibration tests based on an optoelectronic configuration were performed to determine the sensing performance. Additionally, the samples were tested in two different relative humidity environments.

### 2.1 Materials and fabrication

The oxygen sensitive dye of choice was PtOEP (Platinum Octaethylporphyrin, 95%, Merck), purchased as nanopowder, and in turn was encapsulated in the polymer hosts Cellulose Acetate Butyrate (CAB) (MW ∼30,000, Merck) and Polystyrene (PS) (MW ∼230,000, Merck). The dye was embedded into the dense luminescent films by first mixing and sonicating the dye in chloroform (≥99.5%, Merck) and then adding PS and CAB to obtain a final blend with a polymer to solvent w/w ratio of 1:20 (∼4.8 wt. %). The mixture was stirred at room temperature for two hours and then drop cast onto a teflon petri dish to dry out. The dye concentration of each film was kept constant by using the same ratio of dye per polymer; 1:2000 (W/W) (0.05 wt%) with the purpose of having constant amount of dye particles in films of equal weight. This ratio was chosen based on the saturated level of dye dissolved in acetone (which PtOEP has the lowest solubility in). The resulting polymer films were dense with a uniform dye distribution in the centre. The thickness was measured in the centre of each film using a micrometer precise Vernier caliper.

In detail, two different techniques were used to induce porosity to the films; evaporation-induced phase separation (EIPS) and the breath figure (BF) technique. Both are solution based and result in porous films with nano- and macro-porosity throughout the interior of the polymer film. The first relies on the use of solvent/non-solvent combinations in the mixture along with the polymer under study and leads to pore formation due to differences in the solvent and non-solvent evaporation rates [57], [58]. This results in the initiation of the phase separation while the non-solvent creates cavities of varying sizes. The second technique relies on water from the surrounding environment to condense on the surface of the mixture (and later on submerge into it) thus leading to pore formation as the solution solidifies [59]. This is again due to the different rates of evaporation of water and the solvent used.

**Table 1:** Film fabrication characteristics.

Film	Fabrication	Polymer	Solvent	Mixing ratio	Weight range [mg]
A1	Ternary	CAB	Acetone/water	1:8:1	90-150
A2	Ternary	CAB	Acetone/water	1:9:1	90-150
A3	Ternary	CAB	Acetone/water	1:10:1	90-150
A4	Ternary	CAB	Acetone/water	1:11:1	90-150

B	BF	PS	DMF	1:5	150-250
C	BF	CAB	Acetone	1:4	150-250
D	BF	CAB	THF	1:3	250-300

E	Dense	CAB	CHCl${}_{3}$	1:20	90-300
F	Dense	PS	CHCl${}_{3}$	1:20	90-150

It should be mentioned that the coffee ring phenomenon often occurs during the evaporation of drop cast polymer solutions, or colloids in general, and refers to the characteristic shape after the evaporation of the solvent due to the distribution of the solidified material [60]. Here, the coffee ring effect is assumed to remain the same for all films and was neglected. The amount of dye particles per surface area was the same for all cases since the films have the same dimensions apart from their thickness and the experiments are setup such that light is received in the z direction (direction of the thickness) (section S2, Supplementary Material).

The goal of the fabrication process was the development of porous films with a wide range or porosities and pore sizes so that film morphology can then be related to oxygen sensing properties. To this end, porosity control was achieved here with the use of: (i) two different fabrication techniques (BF vs phase separation) (ii) different combinations and ratios of solvents and polymers and (iii) different initial thicknesses of the solutions.

In the first case, ternary solutions were prepared by first mixing the polymer and the solvent/dye into a homogeneous mixture obtained by sonication and then adding the non-solvent. The resulting blend was stirred at room temperature until a ternary (solvent/nonsolvent and polymer) homogeneous solution was obtained. The components in this case were CAB, Acetone (≥99%, Merck) and deionised water, and they were cast in teflon petri dishes to dry out in room temperature. The petri dishes were placed inside a 12 × 12 × 12 cm enclosure in groups of 4 (section S3, Supplementary Material), in order to reduce the evaporation rate, which plays a critical role in the pore formation [61]. Void formation was observed after the drying of the films and specifically hierarchically porous films were expected. The first set of solutions prepared were composed of a polymer/solvent/non-solvent (CAB/Acetone/Water) mixture with a ratio of 1:8:1, 1:9:1, 1:10:1 and 1:11:1. Additionally, for each solution the initial thickness of the mixture in the petri dish was varied in order to change the porosity characteristics [58], [62]. It is to be noted that the increase in initial thickness leads to lower densities [63]. To this end, three different quantities of polymer were used for each solution (see Table 1).

**Fig. 1:**
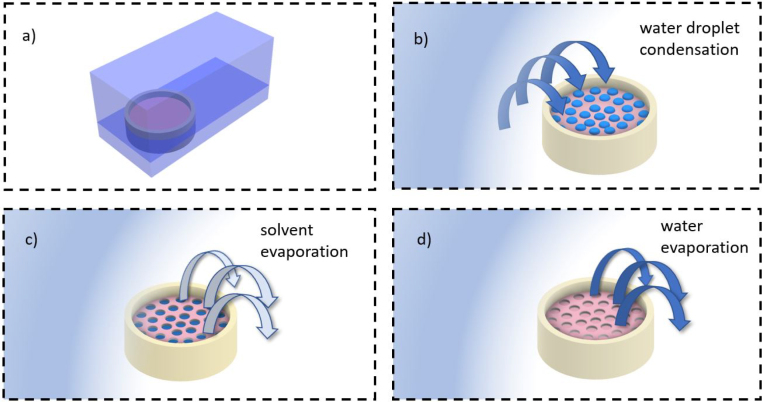
A schematic depiction of the Breath Figure technique in four stages: (a) placement of the initial polymer/solvent solution inside an enclosure with water (b) water begins to precipitate on the surface of the solution (c) as solvent evaporates and the solution becomes viscous the water droplets sink within the film and (d) after the water droplet evaporation the pore formation is complete.

The second fabrication method was based on the BF technique and included the use of PS and CAB as the host polymers [64], [65] (Fig. 1). In this case, binary solutions were prepared from PS/Dimethylformamide (DMF) (≥99.8%, Merck) with a ratio of 1:5, PS/Tetrahydrofuran (THF) (≥99.9%, Merck) 1:4 and CAB/Acetone 1:4 (≥99%, Merck) (Table 1). The resulting solutions were stirred and cast onto teflon petri dishes, left to dry out in a high humidity environment (<90%) at room temperature inside a 12 × 8 × 6 cm enclosure with water (section S3, Supplementary Material). The same dye to polymer concentration was used in all cases once more. However, due to higher viscosity, the amount of polymer for each solution in the petri dishes was higher in this case. This was so that a uniform spatial distribution of polymer was obtained using the same petri dishes (i.e. with equal surface area). For both methods the drying was performed in a fumehood with steady air flow in room temperature.

### 2.2 Instrumentation

For the characterisation of the morphology of the polymer films three imaging techniques were used, namely: SEM (Scanning Electron Microscopy), Digital Microscopy (DM) and Micro Computed Tomography (Micro-CT). The instrumentation used for these measurements was: the Zeiss EVO 25 for SEM, Digital Microscope VHX-7000 by Keyence for DM and Nikon XT H 225 ST for micro-CT, respectively.

On the other hand, the oxygen sensing properties of the dye doped polymer films were analysed based on a standarised calibration procedure with optoelectronic components (Figs. S2.1 and S2.2, Supplementary Material). This consisted of a silicone Thorlabs photodiode with built amplification (DET100A2) as a receiver and an LED (Wurth Elektronik, WL-SUMW Series UV LED, 395 nm, 1100 mW) as the excitation light source. The wavelength of the LED was chosen based on the maximum absorption peak of PtOEP and an optical longpass filter at 610 nm was used in front of the photodiode to only allow light from the phosphorescence emission. The photodiode picks up the reflected light from the sensor film, which is in direct contact with a controlled gaseous mixture of nitrogen and oxygen. Mass flow controllers (El-Flow prestige Bronkhorst) (± 0.01 l/min) were used to control the percentage of each gas and an amperometric device (Hanna Dissolved oxygen meter) was employed to ensure that conditions inside the enclosure were stable. Both transmission and reflection configurations were used due to the expected asymmetry in the porosity and, by extension, sensing performance of the films. The same instrumentation was used in both cases with the difference being the placement of the PD, which was on the other side of the film for transmission tests.

The LEDs were driven by a voltage signal generator (Tektronix AFG106), with a square wave signal of amplitude 3 V (corresponding to 21 mA) and a frequency of 200 Hz, chosen as such so that the decay time does not influence intensity measurements. The output voltage measurements were performed using an oscilloscope (Tektronix TDS 2024C) that was directly connected to the photodiode and the measurements were based on the voltage amplitude of the square wave signal reaching the detector. The components of the system were placed inside an enclosure, an opaque 6 × 6 × 6 cm acrylic box, so that both the photodiode and film were placed inside an environment with high precision oxygen control. The mass flow controllers, the signal generator and the oscilloscope were all controlled using LabView software through a computer. The open source Python Scipy library was used to apply a low-pass Kaiser window finite impulse response (FIR) filter to reduce noise and a non-linear least squares regression to fit the Stern–Volmer plots to the two site model for the half-sensitivity calculations (see section S1 in Supplementary Material for definition).

In order to assess how the humidity influences the oxygen sensitivity of films, a digital humidity sensor (MP780117, Multicomp Pro) was used. Tests were performed in two different relative humidity environments inside the enclosure. High humidity was achieved by using a bubbler, built in situ, using a simple air pump along with a water container. For low humidity, the tests were performed by directly mixing the two gases without the use of the bubbler. The temperature was 23 °C stable within ±0.5°C during the experiments.

### 2.3 Methodology

For the morphological characterisation of the films, both bulk density calculations and imaging techniques were employed. For the density measurements the thickness of each film was measured at the centre and was assumed to be constant; experimentally the variation was within 10% for all cases. The small differences between the sides and the centre of the film were attributed to the coffee ring effect but were assumed negligible.

The first imaging technique was used for the characterisation of the pore morphology in the films. Specifically, digital microscopy was implemented due to the micrometer size of the pores expected from the phase inversion technique (A films). Micro-CT was employed to investigate the asymmetry and the pore distribution in the interior of the films. The images obtained with micro-CT and digital microscopy were processed using the open source software ImageJ and the python implementation of the open library scikit-image. The main objective was pore size calculation and its distribution along the thickness direction. Additionally, SEM images were obtained to discover potential nanoporosity due to a finer resolution possible. This also verified whether the pores were open or closed on the surface, due to the gold coating necessary to capture the images. It is noteworthy that in the SEM images, the pores were identified by looking at the sides of the film after breaking them, which revealed the porosity at the different layers beyond the top surface.

For both the micro-CT and the digital microscopy cases, the first step in the image processing procedure included the transformation of the image into grey scale (section S4, Supplementary Material) and then thresholding (Fig. S4.1a, Supplementary Material), i.e defining the limit above which all pixels are turned to white and below black in order to arrive at a black and white image. For digital microscopy, contrast enhancement and filtering was necessary in order to deduce the correct threshold point (Fig. S4.1b, Supplementary Material). In the case of micro-CT, black related to air and white to the dense material, and the porosity was calculated as a percentage of black pixels over the total (section S4, Supplementary Material). For digital microscopy this was performed in order to focus on specific pore sizes. The contrast enhancement and filtering algorithms (from the Pillow python imaging library) were used in order to focus on the film characteristics of interest, i.e reducing the noise or in some cases ignoring other features such as smaller pores (e.g. in the hierarchical porosity cases). Furthermore, the pore diameter was calculated using ImageJ software.

In terms of oxygen sensing, the main properties investigated were: (a) oxygen sensitivity, S_100_=I${}_{0}/$I_100_ (i.e. ratio of intensity measurements at 100% and 0% of oxygen flow) (b) recovery time, t${↓}_{90}$ (i.e. the time it takes to reach 90% of the maximum intensity value starting from 20% and ending at 0% of oxygen flow) (c) oxygen range and linearity, given as the half sensitivity point $O_{2}\left(S_{max}/2\right)$ (i.e measured here as the point of oxygen V/V where the sensitivity is half of its maximum value) and (d) dynamic range (DR) (measured as the difference in voltage levels between 100% and 0% of oxygen flow) (for detailed calculations see section S1, Supplementary Material).

The oxygen sensing capabilities of each film were assessed by first looking at the recovery time from 0% to 20% of oxygen V/V, set by the mass flow controllers (equivalently 100 and 80% flow of nitrogen). This was also important in order to find out the time period necessary for the intensity of phosphorescent emission to reach a plateau of constant oxygen concentration (section S6 Supplementary Material). Thus, depending on the recovery time, the films were tested sequentially for 8 levels of constant oxygen concentration; 0, 5, 10, 20, 40, 60, 80 and 100% of oxygen flow (Fig. S6.1, section S6 Supplementary Material). It should be mentioned that at 100% the flow was 10 l/min. Moreover, to determine the half-sensitivity point (O${}_{2}$(S=1/2)), the Stern–Volmer plots were fitted with the two site model using least squares fitting with the SciPy Python library.

The amplitude of the signal powering the LED and the distance between the film and the photodiode were kept constant throughout the calibration procedure at 3 V (∼21 mA) and ∼3 cm, respectively. This allowed for a direct comparison of the sensing properties between films of different porosity and their comparison with the non-porous cases. Also, tests were performed for a transmission based configuration for the same distance between film and photodiode, while the LED was placed closer at ∼2.5 cm (due to the size constraints of the enclosure). Both reflection and transmission based measurements were performed in order evaluate the properties of the films in both directions, due to the expected asymmetry in the film formation during fabrication. It is to be noted that both configurations have been employed in the literature depending on the application. Also, the porous films used for transmission measurements were fabricated separately in order to avoid errors due to ageing and photobleaching of the films.

For humidity tests, the measurements were taken for two relative humidity values at the same oxygen concentration; the high relative humidity measurements were performed in air with the use of the bubbler and for the low relative humidity tests the gas tanks were used with a 21:79 ratio V/V of oxygen and nitrogen. The latter procedure lowered the humidity of the enclosure because of the composition of the gases in the tanks. The relative humidity achieved was 10%–15% for the low case and 80%–85% for the high case. It should be noted that the tests were performed for a reflection based configuration in ambient room conditions while the temperature was stable at 22 ±0.5°C.

## 3 Results and discussion

In this section, first the results from the morphology characterisation are presented via bulk calculations and image analysis via Digital Microscopy, Micro-CT and SEM. This is followed by an oxygen sensing characterisation using an optoelectronic configuration and a standard calibration procedure. Additionally, a comparison between transmission and reflection measurements is presented and an overview of the error induced by humidity variation is given. Finally, the relation between film morphology and sensing properties is discussed.

### 3.1 Film morphology characterisation

We determined bulk characteristics and used three imaging techniques to characterise the morphology, namely: Digital Microscopy, Scanning Electron Microscopy and Micro Computed Tomography.

#### Bulk characteristics

The porous films naturally had lower density compared to their non-porous counterparts (for the detailed results see section S5 in the Supplementary Material). Furthermore, the larger weight of the initial solution resulted in lower densities in the case of the A films (e.g. from 0.76 to 0.64 g/cm${}^{3}$ and 0.83 to 0.61 g/cm${}^{3}$, from 90 to 120 mg of CAB, in cases A1 and A2, respectively). This clearly indicates that the pore characteristics change depending on the initial thickness of the A films, as expected (see [66]). Also, beyond a threshold the density stabilised but additional experimentation is needed as higher thickness films were out of the scope of this study due to large recovery times.

However, this was not the case for films fabricated using the breath figure method. Specifically, films C and D exhibited a concurrent increase in the density and weight of the polymer in the solution, whereas for films B, the density remained unaltered. This points towards an asymmetry in the C and D films. An explanation could be based on the condensation of water in layers that resulted in gradients of pore size and porosity that possibly vary with thickness. For films B, it is possible that in order for this effect to influence the bulk characteristics, larger thicknesses are required. Bulk calculations point towards the high porosity of the B films due to lower density values but a more detailed analysis of the film morphology is necessary towards a characterisation of porosity levels and pore sizes. It will be shown later on that gradients of both the porosity and pore size exist along the z axis (Fig. 4).

In the case of the non-porous films, the small density decrease with increasing thickness possibly originated from experimental errors. The predominant explanation would be that this was caused by the coffee ring effect, which was stronger in the case of dense films. This led to the underestimation of the average thicknesses and thus the overestimation of the density (which was more prominent in the films with lower thickness). Between CAB and polystyrene, the latter exhibited lower density.

**Fig. 2:**
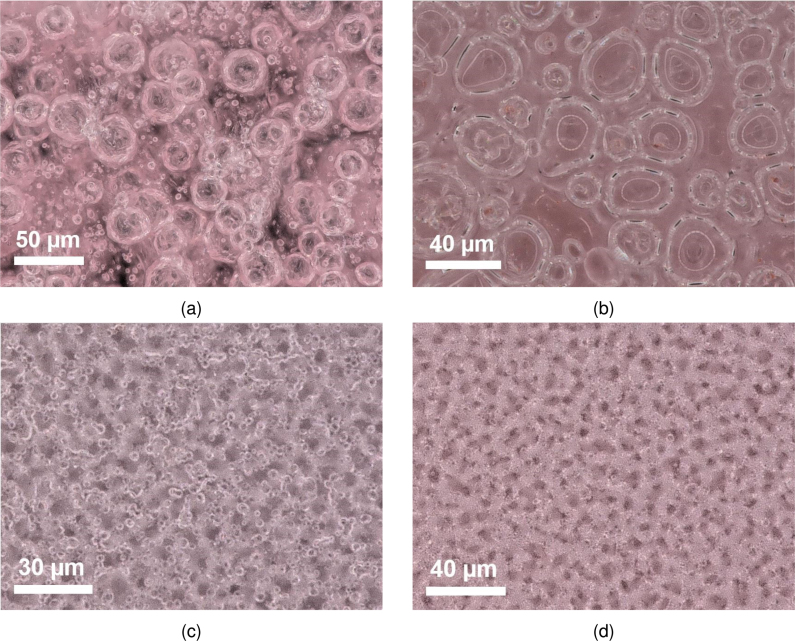
Images of the pores in the films (a) A2.2, (b) B.2, (c) C.2 and (d) D.2 obtained with digital microscopy for the magnification lens ×700.

**Fig. 3:**
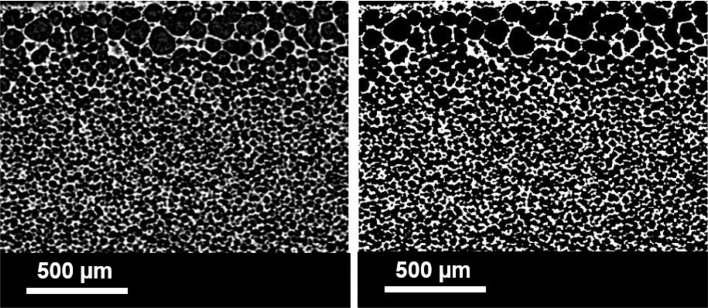
Part of original micro-CT image for a type B film before (left) and after (right) thresholding and contrasting.

#### Image analysis

The first set of images depicting the pore morphology were obtained using digital microscopy (Fig. 2). For the phase inversion technique, film A2.2 was analysed as an example. The hierarchical nature of the porosity was evident due to the existence of macroscale voids (macrovoids) along with smaller diameter pores. This has also been reported in the literature [67], [68]. Fig. S7.1 (Supplementary Material) clearly depicts this by showing film A2.2 at different magnifications and specifically in S7.1b where the camera has focused next to a single macrovoid. Indications of closed pores were also obtained from DM in the case of A2.2. Furthermore, the macrovoid sizes of film A2.2 were determined using imaging analysis techniques (S7, Supplementary Material). Specifically, after applying (a) contrast enhancement and median filtering and (b) thresholding (Fig. S7.2a and S7.2b) the use of the blob detection algorithm was possible in order to determine the position and size of the macrovoids (Fig. S7.2c and S7.2d, respectively). It should be underlined that differences in macrovoid formation varied significantly based on the ratio used for the fabrication of type A films but are not presented here.

For the films based on the breath figure technique, the pores were significantly smaller than the macrovoids and DM did not offer much insight. To be specific, the DM images of film B.2 (Fig. 2(b)) showed that the range of pore sizes is considerably lower compared to that of film A and that the average pore size was much smaller. This can be seen by comparing Fig. 2, Fig. 2, where the magnification was the same. Thus, it was clear that the size of the smallest pores observed in film A2.2 were of comparable size to the ones in B.2. Differences in pore size between the same weight of the B films were not observed or quantified and were considered negligible. Importantly, the surface pores (that can be seen in Fig. 2(b)) could not be categorised as open or closed from digital microscopy. Additionally, image processing using blob detection approach for film B.2 was not possible due to low circularity of the pores and low contrast of the image. Nonetheless, the micropore diameters were identified to range from ∼4 to ∼52 μm. For both films C.2 and D.2 the characteristics observable from digital microscopy (Fig. 2, Fig. 2) showed some similarities between the two. However, at the maximum possible magnification and resolution of the DM equipment, the size of the pores was not discernible for a quantitative analysis. The images obtained via micro-CT for the A2.2 film showed that the pore number inside the films was low and the porosity (defined in Eq. S8.3) was ∼45% with and ∼25% without including the blobs (Fig. S8.1). Moreover, closed pores on the inside of the film were irregularly sized (ranging from ∼77μm to ∼14μm) and randomly distributed along the z direction (see section S8 in Supplementary Material).

**Fig. 4:**
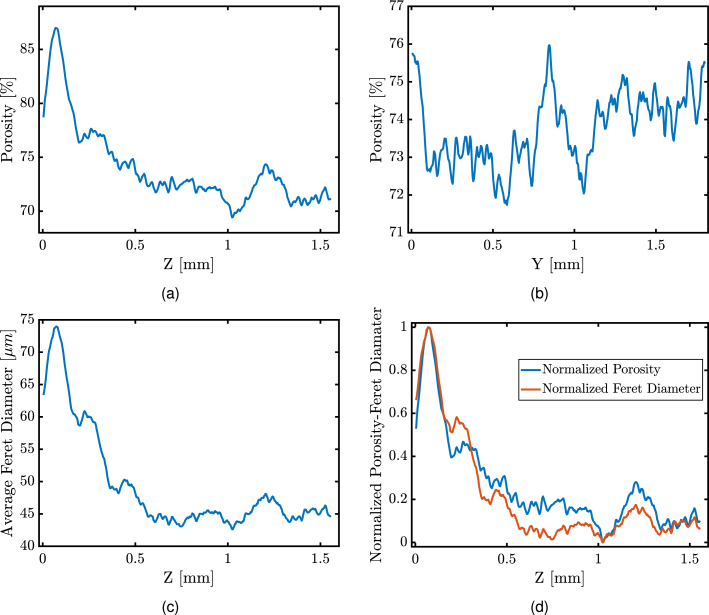
The variation of porosity in (a) z and (b) y direction from the thresholded micro-CT imaging of type B film. (c) Average Feret diameter of the pores versus the thickness (z direction) of the thresholded micro-CT image for film B and (d) normalised porosity and Feret diameter over the thickness (z direction). The plots have been filtered using an FIR filter to reduce noise.

For film B.2, micro-CT was used for the analysis of the pore size and porosity distribution (Fig. 3). Depending on the film and the settings of the micro-CT system, the maximum resolution for the detection of morphological features was ∼1–10 μm. It should be underlined that due to the low density of the film and the small pore sizes a strong contrast (between air and the film) for the pore detection was difficult to achieve. Thus, the analysis for film B involved first the conversion to greyscale and then thresholding (section S8, Supplementary Material). Original and processed images are presented in Fig. 2. The threshold was determined using the isodata point (defined by Eqs. S8.1 and S8.2). The range of porosities calculated was 69%–87% (average porosity of ∼78%) and the pores detected ranged from 10 to 140μm (average pore size of ∼48μm). The measurements presented neglect the Feret pore diameters smaller than 2 pixels (corresponding to less than 10μm). It should be emphasised that the porosity (Fig. 4, Fig. 4) and the Feret diameters (Fig. 4(c)) were calculated along one axis at a time with iterations of 1 pixel.

The results in Fig. 4, Fig. 4 clearly indicate a gradient for both porosity and pore size from top to bottom layers of the film. This asymmetry was expected due to the use of the breath figure fabrication technique [69]. From Fig. 4(d) it was clear that the level of porosity coincided with the size of the pores along the z direction, i.e the larger the Feret pore diameter the larger the percentage of total porosity (section S8, Image analysis: micro-CT). From Fig. 4(b) it was clear that the variations in porosity were not as prominent in the y direction; no clear trend become apparent other than verifying the slight curvature of the film (Fig. S8.2) and the lack of an orderly distribution from this type of fabrication. Note that the regularity of pores was not assessed, but the images presented here resembled the equivalent presented elsewhere for interfacial tensions of ∼40 mN/m [70].

Lastly, SEM was used for all films but was especially important for C.2 and D.1 due to the size of their pores. This enabled higher magnification images and verification of whether the pores were closed or open. In all cases apart from film A2.2 the top surface was comprised of closed pores (opaque image with SEM) whereas on the sides (after breaking the films) the porosity was clearly observable. It should be underlined that the porosity and pore size gradients were not quantifiable using SEM images.

**Fig. 5:**
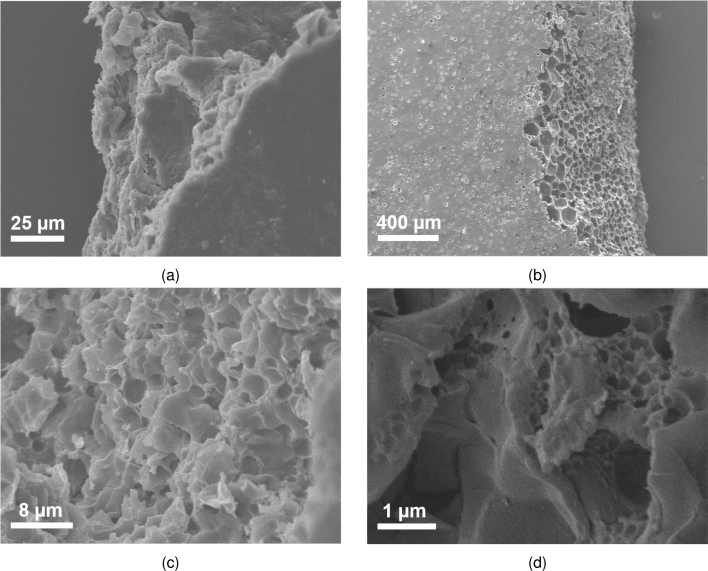
Images obtained with SEM for films (a) A2.2, (b) B.2, (a) C.2 and (b) D.1.

In detail, for film A2.2 the pores were not clearly identifiable but cavities up to ∼54μm wide were seen (Fig. 5(a)). The nature of the morphology of A2.2 seems to be characterised by low porosity as expected from the images obtained via micro-CT. For film B2.2 the large size of the pores as well as the high levels of microporosity allowed their clear depiction from SEM on the sides (Fig. 5(b)). The pore sizes observed ranged from ∼37 to ∼141μm but it should be underlined that their identification throughout the thickness of the film was not possible with SEM. Additionally, small diameter holes (ranging from ∼35 to ∼11μm) were observed on the top surface, but were infrequent and randomly distributed.

For film C.2 the pore sizes ranged from ∼0.47 to ∼3.42μm (Fig. 5(c)) which would also explain the inability of the DM to obtain a clear depiction of the pores (Fig. 2(c)). The morphology of the surface analysed exhibited many similarities to film A2.2. For film D.1, pores with a diameter ranging from ∼37 nm to ∼1.07μm were observed (Fig. 5(d)). This range was expected as smaller pore sizes have been reported with the use of THF as a solvent in combination with PS while employing the breath figure technique in the literature [71] and the indications from digital microscopy presented earlier (Fig. 2(d)).

### 3.2 Oxygen sensing properties

The films were tested and compared in terms of: (1) amount of polymer (and thus indicating also the amount of dye present in each film and the initial thickness of the solution), (2) thickness, (3) density, (4) maximum sensitivity, (5) recovery time, (6) half-sensitivity point and finally (7) dynamic range (see section S1, Supplementary Material for definitions).

To begin with, a direct link between densities and recovery times was not found when comparing all the films (for a detailed description of the results see section S9, Supplementary Material). Nonetheless, a key finding was that type B films, which had significantly lower densities compared to the rest, had smaller values of recovery time by an order of magnitude. Importantly, the recovery time of type B films seemed to stabilise from B.2 to B.3 and any further increase in thickness did not result in a rise of recovery times. This has not been shown previously in the literature. Also, dense polystyrene films exhibited much higher recovery times, as expected due to the higher density of the films which lead to larger diffusion barriers.

In all cases, increasing the amount of polymer led to an increase in S_100_ (maximum sensitivity), $O_{2}\left(S_{max}/2\right)$ (half-sensitivity) and DR of the measurements, as expected due to the increase in the number of dye molecules (the dye concentration was the same in all cases). It should be noted though that unlike the dense films, when using the phase separation approach, the amount of polymer affected the initial thickness which in turn altered the porosities. This was the key reason that the increase in sensing performance was steeper in the case of the ternary solution based films compared to the rest (Tables S.2 and S.3, Supplementary Material). It is to be noted that larger amounts of initial solution in the case of A films produced samples of increasingly larger recovery time and were not considered for the purposes of this study. Nonetheless, for the films prepared with same weight (A1.3, A2.3, A3.3, A4.3), similar S_100_, half-sensitivity points and DR were observed within experimental error. However, upon increase in polymer weight, for example, from A1.1 to A1.3 a sensitivity rise of was 95% was observed. On the other hand, by increasing the amount of polymer the sensing properties did not show a dramatic improvement in the dense films; e.g. from E.3 to E.6 the polymer weight was doubled but the sensitivity increased by 67%. This effect was even more prominent for the BF based porous films; e.g. from B.1 to B.3 the polymer weight increase was 66.7% while the sensitivity remained to a large degree the same (negligible increase by 3%). Thus, this clearly indicates that the fabrication procedure allows for significant increases in sensing capabilities.

**Fig. 6:**
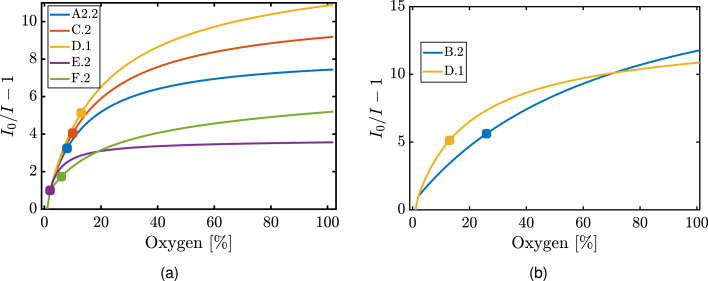
(a) Plots of I${}_{0}/$I${}_{x\text{\%}}-1$ against the oxygen percentage of flow from 0 to 100% V/V for films A2.2, C.2, D.1, E.2 and F.2 after the fitting of the curve with the two site model. The $O_{2}\left(S_{max}/2\right)$ point for each curve is also highlighted. (b) Plots of I${}_{0}/$I${}_{x\text{\%}}-1$ against the oxygen percentage of flow from 0 to 100% for films B.2 and D.1 after the fitting of the curve with the two site model. The half-sensitivity point (O${}_{2}$(S=1/2)) is highlighted for each curve (•).

In total, type B films produced the best overall results such as low density and low recovery times coupled with high maximum sensitivity and dynamic range (see Fig. 6). Additionally, it was observed that film C.1 presented the same S_100_ with the same polymer amount as A4.3 but with a fraction of the recovery time and a higher DR (it should be noted that the half-sensitivity points were equal) (Table S.2). In terms of $O_{2}\left(S_{max}/2\right)$, type B films once more presented significantly better properties than most of their counterparts, indicating that the sensitivity was higher at higher oxygen concentrations compared to the rest of the films.

To analyse the improvement yielded by the fabrication techniques employed, a comparison of the sensing performance between the dense and porous films is presented in terms of: the relative changes in the maximum sensitivity ($\Delta S_{100}/S_{100}^{d}$, Eq. S9.1), the recovery time ($\Delta t{↓}_{90}/t{↓}_{90}^{d}$, Eq. S9.2) and dynamic range ($\Delta \left(DR\right)/DR_{d}$, Eq. S9.3) along with the absolute change in the half-sensitivity point ($\Delta O_{2}\left(S=S_{max}/2\right)$, Eq. S9.4) (see Table 2). It is to be noted that the non-porous film against which the porous sample is compared to, was chosen based on the type of polymer and its weight where possible (e.g. B.1 was compared with F.2 as the closest choice of reference).

In detail, by comparing dense (i.e. non-porous) and porous films (Table 2) it is evident that the latter produced better results in terms of S_100_, $O_{2}\left(S_{max}/2\right)$ and DR. Noticeably, despite the lower densities of the type A films compared to their dense counterparts, higher recovery times were observed. This can be attributed to: (i) lower porosity (ii) higher thickness and (iii) low surface area of the films due to the existence of the large macrovoids (blobs, Fig. S8.3). Thus, an important result was that the size of the pores and the porosity distribution greatly affect the recovery times. In detail, for reflection based measurements, the best film developed with the phase separation methodology (A4.3) showed 119% increase in maximum sensitivity, 8 $O_{2}\left(S_{max}/2\right)$ % in half-sensitivity point, a factor of 3.1 in DR from their counterpart CAB dense film (E.3) followed though by a factor of 5.08 increase in recovery time (Table 2).

**Table 2:** Comparison of the oxygen sensing properties between the dense and porous films for the reflection based setup.

Film	$\Delta S_{100}/S_{100}^{d}$ [%]	$\Delta t{↓}_{90}/t{↓}_{90}^{d}$	$\Delta O_{2}\left(S=S_{max}/2\right)$ [%]	$\Delta \left(DR\right)/DR_{d}$
A1.3	94.51	−3.54	8	1.93
A2.3	93.56	−2	7	1.93
A3.3	86.16	−2.31	6	1.48
A4.3	118.62	−4.08	8	2.10
B.2	128.65	0.92	15	6.92
C.2	85.63	0.17	7	1.76
D.1	72.84	0.10	8	0.81

On the other hand, with the use of the BF method, C.2 and D.1 exhibited an increase of ∼86% and ∼73% in maximum sensitivity, a 7 and 8% V/V in half-sensitivity and 176%, 81% (0.7, 0.43 V) in DR, compared to their counterpart dense CAB film (E.5). Moreover, when comparing dense and porous films with the same weight using polystyrene (F.2 and B.1), an increase of a factor of 2.14 in sensitivity, 15% V/V in half-sensitivity point and factor of 7.3 (1.64 V) in DR was observed, while the recovery time was reduced by 92%.

**Fig. 7:**
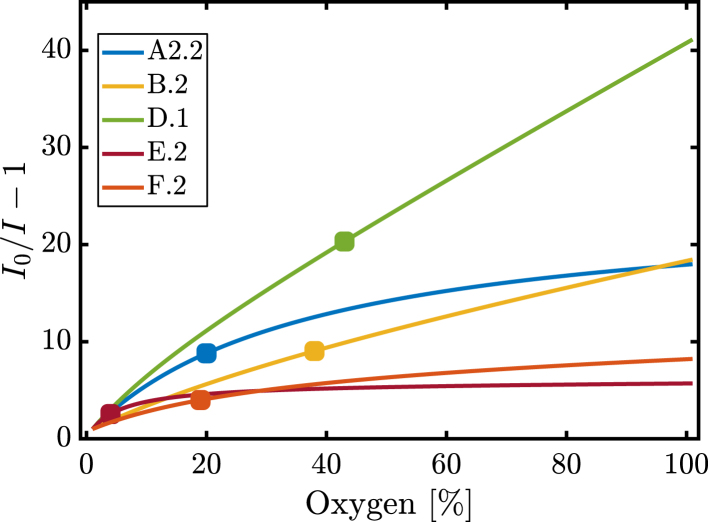
Variations of I${}_{0}/$I${}_{x\text{\%}}-1$ against the oxygen percentage of flow from 0 to 100% V/V for films A2.2, B.2, D.1, E.2 and F.2 after the fitting of the curve with the two site model for the transmission based configuration. The half-sensitivity point (O${}_{2}$(S=1/2)) is highlighted for each curve (•).

The second part of the oxygen sensing characterisation was performed with a transmission based configuration. By comparing results between transmission and reflection based setups it is evident that the latter presented much lower S_100_, $O_{2}\left(S_{max}/2\right)$ and DR values (Tables 3, 4 and Fig. 7). Although there was an increase in luminosity reaching the film due to the differences in the optical setups, this is not sufficient to explain the large differences observed in the sensing properties between the two configurations. In detail, estimates based on irradiance calculations showed an ∼18% increase in irradiance onto the film from a reflection to a transmission based setup. However, experimentally, the maximum voltage observed for each film was significantly different. This was attributed to variations in the phosphorescence emitted and subsequently captured by the PD; a maximum of an ∼9.5 fold increase in voltage was measured between the two cases, at 0% oxygen V/V. The large increase in DR was a clear indication that irradiance differences between the two set-ups are insufficient to explain the large increase observed for the same films under the same oxygen concentration. Thus, the differences were attributed to both the asymmetry in the pore formation [72] and the different light diffusion properties of the films in relation to the direction of the excitation light. To this end, it is expected that thickness, pore size and porosity (and their distribution) were all contributors to this effect.

Consequently, contrary to the reflection based measurements discussed previously, C.2 and D.1 presented significantly improved sensing capabilities in terms of S_100_, $O_{2}\left(S_{max}/2\right)$ and DR compared to the other films (Table 3). Additionally, there was no universal relation established between the sensing capabilities of the films in a transmission mode compared to a reflection mode setup, e.g. A3.2 performed worse compared to the rest of the ternary based films for the same amount of polymer and at the same time B.2 performed worse than A4.2. However, for the breath figure technique, it was obvious that type B films exhibited poorer sensing properties in transmission. Hence, it is clear that there exists a preference for the use of different porous films in each measurement mode; type B films are suited for reflection based and D for transmission based measurements.

**Table 3:** Oxygen sensing properties obtained from the transmission setup measurements.

Film	Thickness [μm]	Density [g/cm${}^{3}$]	I${}_{0}$/I_100_	t${↓}_{90}$[s]	O${}_{2}\left(S=S_{max}/2\right)$ [%]	DR [V]	
A1.2	158	0.71	16.96	120	19	8.05	
A2.2	169	0.66	18.17	100	20	7.84	
A3.2	164	0.69	13.15	72	14	5.56	
A4.2	175	0.64	19.16	144	21	8.82	
B.2	874	0.22	18.63	24	38	5.67	
C.2	160	1.16	33.45	152	35	11.67	
D.1	205	1.13	42	184	43	11.35	
E.2	80	1.40	5.73	24	4	3.05	
F.2	98	1.14	8.24	276	19	4.90

**Table 4:** Comparison of the oxygen sensing properties between the dense and porous films for the transmission based setup.

Film	$\Delta S_{100}/S_{100}^{d}$	$\Delta O_{2}\left(S=S_{max}/2\right)$ [%]	$\Delta DR/DR_{d}$
A1.2	1.96	15	1.64
A2.2	2.17	16	1.57
A3.2	1.29	10	0.82
A4.2	2.34	17	1.89
B.2	1.26	19	0.16
C.2	4.84	16	2.83
D.1	6.33	24	2.72

**Table 5:** Table comparing the improvement in sensing performance via polymer film modification from various studies in the literature and the films proposed here.

Polymer	Dye	Metric	Improvement	Fabrication	Reference
PS/PEG	–	Luminescent Intensity	60%	Dropcasting	[42]
pSiMC	EuA	Luminescent Intensity	100%	Etching	[73]
MTEOS	R(Ph${}_{2}$phen)${}_{3}^{2+}$	$\Delta \left(DR\right)/DR_{d}$	73%	Drop casting	[74]
MTEOS/TEOS	RuCl${}_{3}$3H2O	$\Delta \left(DR\right)/DR_{d}$	92%	Dropcasting	[75]

CAB (C.1)	PtOEP	$\Delta \left(DR\right)/DR_{d}$	2.8	Breath Figure	–

PS/PEG	PtOEP	$\Delta S_{100}/S_{100}^{d}$	91%	Dropcasting	[41]
PS/PG	PtOEP	$\Delta S_{100}/S_{100}^{d}$	72%	Phase Separation	[39]
PDMS-MPA	PtTFPP	$\Delta S_{100}/S_{100}^{d}$	30	Photo-lithography	[49]

CAB (D.1)	PtOEP	$\Delta S_{100}/S_{100}^{d}$	6.3	Breath Figure	–

Table 4 summarises the improvement achieved for a transmission based set-up. The best case of ternary based film (A4.2) showed a factor of 3.34 increase in the sensitivity compared to E.2. Equivalently, for the BF technique and CAB as the host polymer, D.1 showed an improvement of a factor of 7.33. In the case of polystyrene as a host polymer, B.2 presented a 2.26 fold larger sensitivity compared to F.2. The respective changes for the DR obtained were: a factor of 2.89 for A4.2, 3.72 for D.1, 16% for B.2 and 3.83 for C.2. Furthermore, the increase in DR when comparing porous and dense films ($\Delta DR/DR_{d}$) was a maximum factor of 3.8 and 7.9 for transmission and reflection based setups, respectively. The equivalent change in sensitivity was a maximum factor of 7.33 and 2.26 and the linearity of the Stern–Volmer curves was also improved by 15 and 24% V/V.

For a comparison with the literature, the transmission based measurements should be the reference point since the luminescent intensity reaching the film was significantly lower for the reflection based tests, when compared to most studies. Given this, the porous films proposed here showed significantly higher improvements to sensor performance (Table 5). For example, in the studies by Lee et al. [39], a maximum of 72% increase in sensitivity was observed by using phase separation techniques to induce porosity in polystyrene films.

### 3.3 Humidity effects

Humidity is known to influence the sensing performance [43]. In particular, the relation between oxygen measurements and relative humidity was investigated for both porous and dense films. This was carried out to verify whether the films proposed here are affected by humidity changes of their environment and compare their performance to their dense counterparts. The tests were performed with a reflection based configuration for two different humidity levels (see also section S10, Supplementary Material).

The largest induced error was seen on film A2.2 and the lowest in D.1. It should be noted that the dense film of polystyrene showed the smallest error compared to all films and that the breath figure method showed better results compared to the phase inversion technique. This was possibly the result of the larger macrovoid pores found with the latter and their lower sensitivity. Nonetheless, the error introduced was small and comparable to the dense films in all cases. Also, D.1 and C.2 presented a smaller variation than the CAB dense film. This is either due to the increased sensitivity of the films to oxygen (the percentage shown is a relative comparison) or possibly due to an additional hydrophobicity of the porous films. Further studies are required to investigate the latter. A more detailed account of the calculations is given in Table S.4 in section S10 of the Supplementary Material.

Overall, the relative humidity tests showed that the film fabrication methodologies did not induce significant error in the oxygen sensing capabilities while the best films corresponded to the BF technique and the use of CAB as a polymer.

### 3.4 Discussion: Morphology and sensing performance

By relating the oxygen sensing capabilities of the films with their morphological characteristics, some general observations can be made. First, by comparing the two fabrication procedures; the breath figure technique produced a wider range of bulk densities, pore sizes, porosities and surface morphology variations while simultaneously achieving the highest improvement in terms of their oxygen sensing properties. For a reflection based methodology, type B films yielded the best oxygen sensing performance and exhibited the highest porosity. In particular, the most noticeable advantage was the lower recovery time which was reduced by an order of magnitude. It is clear that the increased porosity and low thickness of the dense parts within the film contributed greatly towards the lowering of the recovery time. Incidentally the overall film thickness does not influence this noticeably. This was in part expected; in fact recovery times have been shown to be related to the thickness by a power law (for dense films) [76] and the pore walls essentially control the diffusion in the porous films investigated here. In separate experiments, it was observed that the density and the recovery time were stable (±10%) up to a thickness of ∼1.7 mm.

It should be underlined that this is an outcome of crucial importance since the thickness of type B film does not need to be restricted in order to achieve low recovery times. This is not the case in dense and porous films fabricated with other methodologies and has not been shown for a solid film in the literature, to the best of our knowledge. By extension, this could lead to much higher sensitivities for indicators and oxygen sensing devices where the limiting factor was the long recovery time; either due to the need (of a high thickness film) for a greater amount of dye particles or increased robustness in terms of their mechanical properties. However, it should be noted that the maximum sensitivity also saturated for thicknesses greater than that of film B.2. This suggests that more detailed studies are required to evaluate the potential benefits of a further increase in thickness for type B films in terms of oxygen sensing performance. Also, doubling the dye concentration produced minimal changes in the film’s performance. This is an indication that the dye concentration used for the B films was in the saturation region in terms of concentration of dye versus output performance (which was not the case for the dense films). Additional studies though are required to verify this.

A key remark should also be that the total surface area (SA) inside the total film is considered to play a crucial role since oxygen diffuses through the entirety of the film. To consider what happens in the external surface of the film, by neglecting diffusion into the deeper layers, additional calculations are required or the use of another technique for SA calculations (e.g. Brunauer–Emmett–Teller (BET) adsorption tests). Based on bulk calculations of the volume change between the B.1 film and its dense closest counterpart (F.3), a ∼32.3 fold increase in the total surface area is expected if we assume that the average pore size is the same for B.1 and B.2 (see section S11, Supplementary Material).

Furthermore, an important result from the use of the films proposed in this study was the improved linearity of the Stern–Volmer calibration curve. This was described here with the half-sensitivity point and dynamic range, which essentially translates into higher relative sensitivities in the high oxygen concentration regions. This clearly shows the versatility of the porous films proposed for monitoring a wider range of oxygen concentrations. Notably, this could allow the universal use of dyes that have a short range of high performance but saturate considerably in high oxygen environments (such as PdOEP).

Moreover, it has been shown [39] that surface morphology plays a greater role for oxygen accessibility and thus improves sensitivity proportionally more. Nonetheless, the results presented in this study show that much higher sensitivity improvements were possible even when retaining a thin dense layer on the surface facing the input light (such as in type B films). This was evident from the dense/opaque “barrier” seen on the top and bottom surfaces of the films but porous morphology on the sides (see Fig. 5). However, the dense outer layer could improve the mechanical properties of the film and thus a trade-off might be called for. Additional studies are required to investigate this systematically and are beyond the scope of the current work.

Finally, a novel result presented in this study is the variation of sensing properties with respect to the placement of the light detector. This became apparent when comparing the two configurations: transmission and reflection based. Notably, the type B films yielded the worst performance compared to the other films for transmission based measurements but the most favourable for reflection based. This was attributed to the different reflectance and transmittance of the films as a result of their different optical diffusion and scattering properties (section S12, Supplementary Material).

In essence, the opaqueness and reflectance of type B films benefited the sensing properties in one case but were detrimental for the second. On the other hand, type D and C films significantly increased their sensing output with transmission measurements. Moreover, type D and C films had the largest density and thus lowest thickness. This points out that low thickness films in combination with smaller pores constitute ideal dye host supports for transmission based measurements. Nonetheless, the drawback of high recovery times persisted (with the exception of C.1). Subsequently, the fabrication of nano-porous films with high porosity and low thickness would be optimal. Further studies should focus on separating the contributions towards improved performance, due to light scattering and increased permeability.

## 4 Conclusions

In this study, an oxygen sensitive dye was embedded in PS and CAB by using self-assembly techniques to enhance the sensing performance. Specifically, porosity was introduced via two different solvent-based fabrication techniques using drop casting, namely; phase separation of ternary solutions and the breath figure technique. Two different polymers, CAB and polystyrene, and three different solvents (THF, DMF and acetone) were employed to fabricate four different types of films with different porosity characteristics. The films developed were compared with their dense counterparts to directly assess the improvement in sensing performance. The experimental methodology included a morphological investigation, based on image analysis, to relate specific structural characteristics to sensing properties. This was followed by the use of a standardised calibration procedure to determine oxygen sensing properties, for both transmission and reflection based optical configurations. The effect of the variation of the humidity on the sensing performance was also shown.

The main findings include: (i) The breath figure technique was the preferred choice among the two fabrication methods due to a wider variety of morphologies produced and a larger improvement in sensing performance; pore sizes ranged from ∼37μm to ∼141 nm and an increase of a factor of ∼7.9 in dynamic range was achieved. (ii) High porosity and small pores were linked to improved sensing. Notably, with the Breath Figure method, the combination of THF and CAB (type D films) displayed the smallest pore sizes and DMF and PS (type B films) the largest porosity. (iii) Different film morphologies were preferable depending on the configuration: transmission versus reflection based. (iv) For a reflection based setup, the combination of polystyrene and DMF (type B films) using the breath figure technique exhibited the most favourable characteristics in terms of: maximum sensitivity (∼12), dynamic range (2.06 V), half-sensitivity point (27% V/V) and recovery time (8 s). This was accompanied by the lowest density, ∼0.22 g/cm${}^{3}$. (v) For transmission based measurements and using the breath figure technique, the mixture of THF and CAB (type D film) yielded the best results with a maximum sensitivity of ∼42, dynamic range of 11.67 V and half-sensitivity point at 43% V/V. (vi) Comparing the two types of configurations; the transmission based setup resulted in a larger increase in maximum sensitivity (factor of ∼7.9 versus ∼2.3) and half-sensitivity point (24% versus 15% V/V). (vii) The sensing properties of the porous films were not appreciably affected by humidity.

On the whole, the implementation of self-assembly fabrication techniques based on the evaporation of ternary solutions and the Breath Figure method produced a wide range of pore sizes and yielded significant improvements in sensing properties. It should be emphasised that key benefits include the use of widely available materials and straightforward fabrication procedures, accomplished via drop casting and solvent evaporation. In parallel, an image based analysis of the porosity allowed for a detailed investigation of the resulting morphology and its relation to output sensing properties. Thus, critical insights into the preferable material composition and morphological structure of the polymer supports could be gained. In turn, this provides a novel approach to polymer modification for phosphorescent dye encapsulation and opens the road for commercial availability.

Further investigation is suggested for the determination of the optical characteristics of the films with and without the phosphorescent dyes. This would demonstrate and separate the contributions towards improving oxygen sensing from the enhanced optical properties and the increased permeability of the films. Additionally, the use of BET surface calculations should be performed for a detailed characterisation of the films’ surface area. This could point towards a waveguide approach for oxygen sensing with porous materials and hence combine (i) an increase in oxygen permeability and (ii) increase in light output. This could potentially lead to a wider variety of sensing capabilities for all types of luminescence-based devices.

## CRediT authorship contribution statement

**Nikolaos Salaris:** Conceptualization, Methodology, Software, Validation, Formal analysis, Data curation, Writing – original draft, Writing – review & editing, Visualization, Investigation. **Paul Haigh:** Supervision, Writing – review & editing. **Ioannis Papakonstantinou:** Conceptualization, Supervision, Writing – review & editing. **Manish K. Tiwari:** Conceptualization, Supervision, Writing – review & editing, Project administration.

## Declaration of Competing Interest

The authors declare that they have no known competing financial interests or personal relationships that could have appeared to influence the work reported in this paper.

## Data Availability

Data will be made available on request.

## Appendix A Supplementary material

MMC S1
