# Supplementary material for: Self-assembled porous polymer films for improved oxygen sensing
Source: Sens Actuators B Chem. 2023 Jan 1;374:132794. doi: 10.1016/j.snb.2022.132794 (PMC10582206; doi:10.1016/j.snb.2022.132794)
Supplement: MMC S1 [file mmc1.pdf]

Supplementary Material

Self-assembled porous polymer films for improved oxygen sensing

Nikolaos Salaris[a,b], Paul Haigh[c], Ioannis Papakonstantinou[d], Manish K. Tiwari[a,b,*]

[a] Nanoengineered Systems Laboratory, UCL Mechanical Engineering, University College London, London WC1E 7JE, United Kingdom
[b] Wellcome/EPSRC Centre for Interventional and Surgical Sciences (WEISS), University College London, London W1W 7TS, United Kingdom
[c] School of Engineering, Newcastle University, Newcastle, NE1 7RU, United Kingdom
[d] Photonic Innovations Lab, Department of Electronic & Electrical Engineering, University College London, London WC1E 7JE, United Kingdom

S1 Definitions

a) Dynamic range (DR) is defined as the difference between the measurements at the two different oxygen levels (eq. S1.1). In this study, the two oxygen concentrations were 0 and 100 % V/V.

$$DR = I_0 - I_{x\%} \tag{S1.1}$$

where the underscores 0 and $x\%$ refer to the oxygen content in the environment under study (V/V in the case of our experiments).

b) The sensitivity is defined as the ratio of the measurements at two oxygen levels, either intensity of lifetime, where the numerator is the 0% V/V oxygen level and the denominator a specific oxygen concentration (eq. S1.2). In this study, the two oxygen concentrations were 0 and 100 % V/V (S_{100}), i.e. the maximum possible sensitivity.

$$Sensitivity = S_x = \frac{I_0}{I_{x\%}} \tag{S1.2}$$

where x% is the percentage of oxygen content in the environment under study.
c) One method to characterize the calibration curve of an oxygen sensing device in terms of the range of oxygen measurements analysed is the use of the half-sensitivity point. This is defined as the oxygen point where the sensitivity is half of the maximum; i.e $O_2(S = S_{max}/2)$ or else 3dB point (eq. S1.3):

$$S_{O_2(S=S_{max}/2)} = \frac{S_{100} - S_0}{2} \tag{S1.3}$$

d) To characterize the time it takes for the oxygen sensor measurements to reach a stable point; the terms response/recovery times are used in order to indicate the time necessary for the stabilisation of the measurements in an equilibrium state coming from lower/higher oxygen concentrations. These two values are defined as follows: the response/recovery times are $t \uparrow_{90\%}$ and $t \downarrow_{90\%}$ respectively, i.e as the time necessary for the measurements to reach 90% of the stabilised oxygen plateau value for a constant oxygen concentration.

Additionally, a general observation was that the dynamic range given at a specific oxygen point represents the total accumulated changes from one oxygen concentration to the next (by means of difference in voltage in this case) rather than the sensitivity of the sensor presented only at that point. This is due to the Stern-Volmer equation used in the literature for phosphorescence based oxygen sensors which linearly relates the inverse of the measured quantity (in this case the intensity of light reaching the detector) to oxygen concentration. Thus, the sensitivity of these sensors is measured in ratios rather than differences. It is the author's opinion that the DR allows for a more representative value of the sensor's performance over a range of oxygen concentrations whereas the sensitivity provides targeted information about the sensor's output in a specific concentration.

* Corresponding author, email: m.tiwari@ucl.ac.uk, phone: +44 20 3108 1056, postal address: Room 504C, Roberts Building, Torrington Place, London, WC1E 7JE

S2 Experimental Configuration

In this section, schematics of the test setup (Fig. S2.1) and the LED positioning (Fig. S2.2) along with images of the experimental set-up and mass flow controllers (Fig. S2.3) are presented. In particular, for the case of the setup schematic, the positioning of the LED, amperometric sensor, silicone sealing, photodiode, dye doped film and optical filter are highlighted. In Fig. S2.2 the positioning of the dye doped polymer films in relation to the LED is highlighted. In Fig. S2.3a, an image of the setup which shows the signal generator, oscilloscope, gas tanks, amperometric oxygen sensor, testing enclosure and the computer controlling the equipment is presented. A close up image of the mass flow controllers is also shown in Fig. S2.3b.

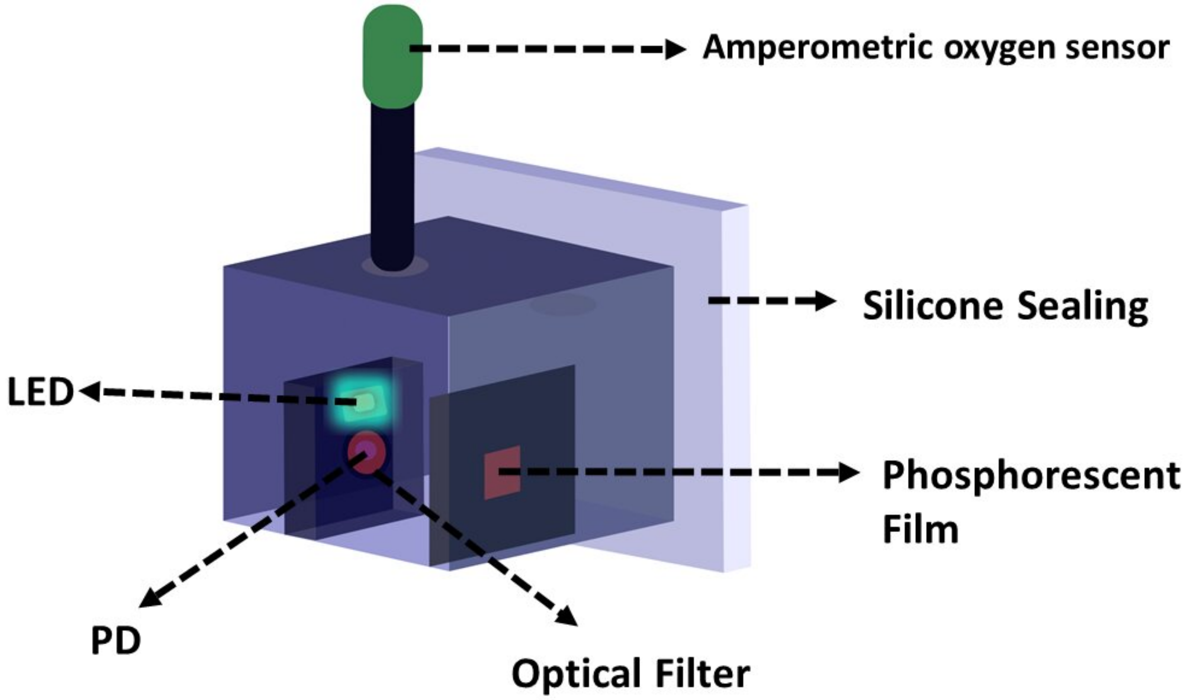

Fig. S2.1: Schematic of the test setup.

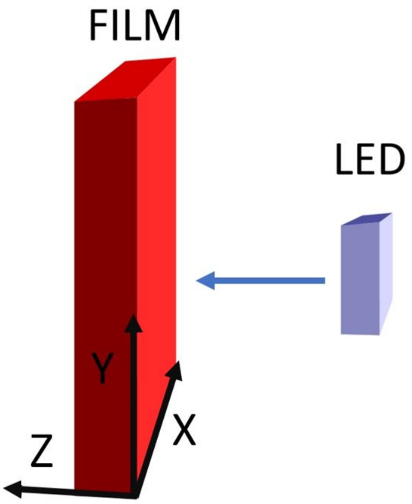

Fig. S2.2: Schematic of the film and LED positioning

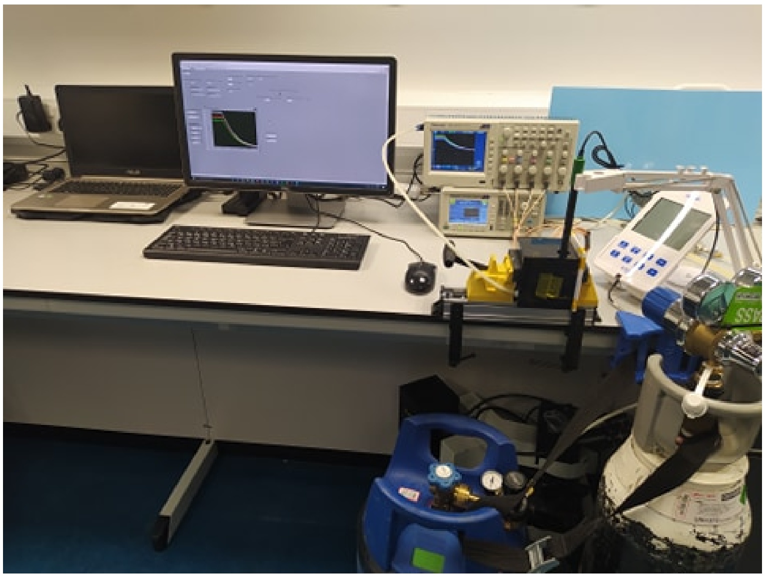

(a)

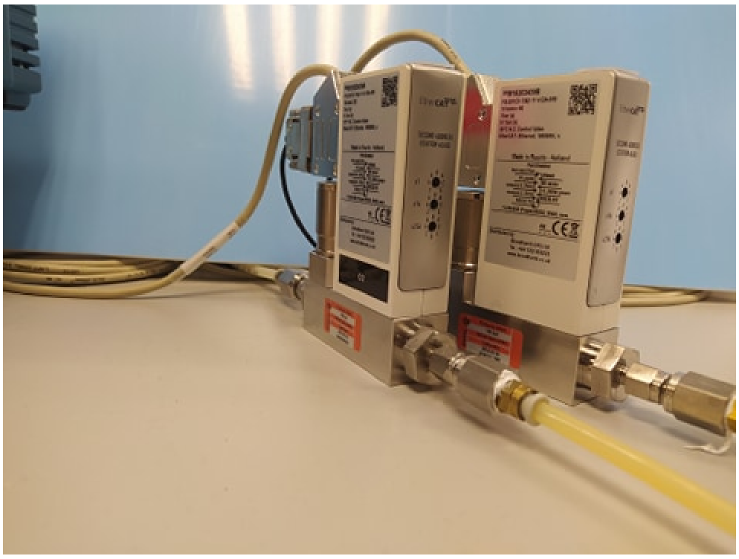

(b)

Fig. S2.3: (a) Images of the instrumentation and their configuration and (b) the mass flow controllers.

S3 Fabrication Schematics

In Fig. S3.1, the schematics of the fabrication procedure are presented. In detail, the homogeneous solutions are first dropcasted into teflon petri dishes and then placed in an enclosure which in turn is placed on top of a hot plate and into a fumehood. For the Breath Figure method, the humidity is increased with the addition of water in the enclosure. In all cases, the air flow is kept constant by having specific holes on the walls of the enclosure while the velocity of the air flow is monitored by the fumehood. All the different components are highlighted in the schematic.

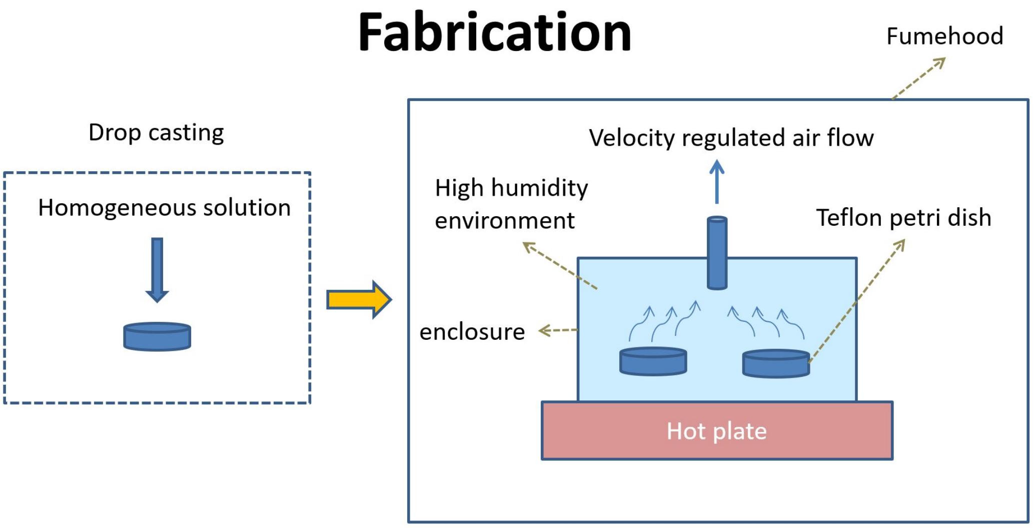

Fig. S3.1: Schematic of the film fabrication procedure followed for the breath figure method.

S4 Image Processing

In this section, part of the image processing procedure followed for the morphology characterisation of the porous films is presented. First, the equation used for transforming the colored images to grayscale is shown (Eq. S4.1). Then a graph plotting the number of pixels (N) over the colorscale (0 to 255) for the grayscaled image of A2.2 (for a magnification lens of $\times$ 20) is presented as an example, while the threshold is indicated with a red dashed line (Fig. S4.1a). Secondly, the same plot of number of pixels over colorscale for the contrasted image is displayed, while once more the threshold is indicated with a red dashed line (Fig. S4.1b). Notably, this procedure is the necessary image analysis preceding the use of the blob detection shown further on in Fig. S7.2d in order to reach at Fig. S7.3.

$$Y = 0.2125 \times R + 0.7154 \times G + 0.0721 \times B \tag{S4.1}$$

where R, G and B are the red, green and black pixels respectively.

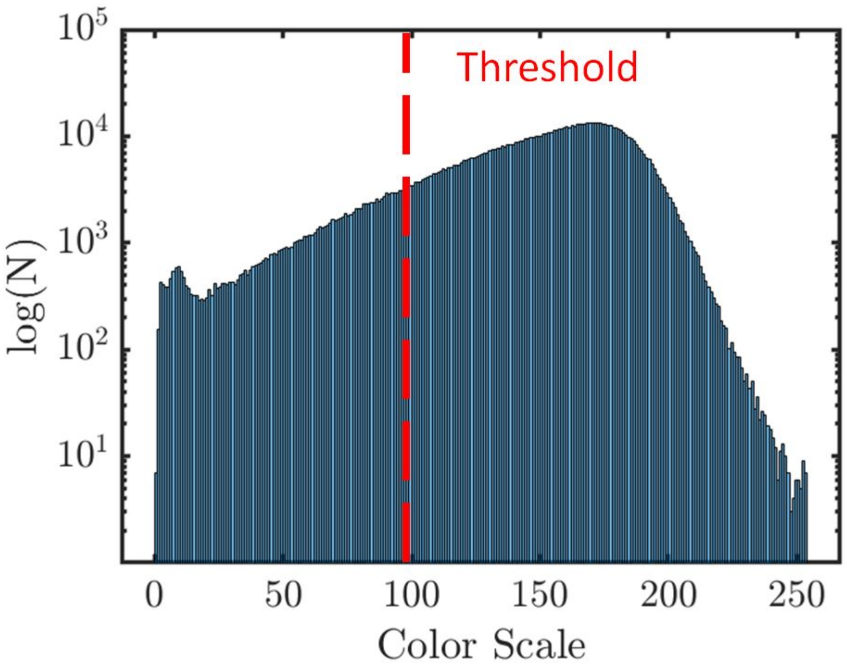

(a)

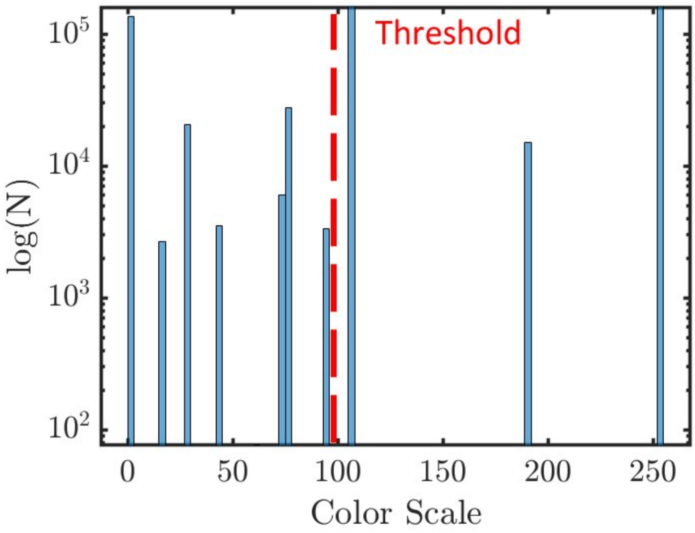

(b)

Fig. S4.1: Color histogram of a) grayscaled image of A2.2 for the magnification lens ×20 where the final threshold used is displayed with a red dashed line and b) the contrasted image of A2.2 for the magnification lens ×20 while the threshold used is indicated with a red dashed line

S5 Bulk Calculations

The bulk caculations of all the films produced and tested (both porous and dense, i.e. non-porous) are presented in Table S.1. The film notation (e.g. A2.3) is based on: a) the fabrication methodology (e.g A films) b) the ratio of the mixture where applicable (e.g. A2 films) and c) the amount of polymer (A2.1 - A2.3 films). This is also explained in the main part of the paper in section 2.1 Materials and Fabrication (Table 1). Additionally, the amount of polymer, the thickness and the density of the films are indicated. These measurements were performed with a scale and a micrometer precise digital Vernier Caliper.

Table S.1 presents the bulk calculations and provided the first indications of the different film porosities due to i) the density variation for different polymer/solvent ratios and ii) the two different fabrication techniques. Another key outcome was the change in densities depending on the amount of solution in the petri dishes in most cases, with the exception of the type B films. Importantly, the dense films yielded significantly higher densities compared to the porous films, as expected.

Film	Polymer [mg]	Thickness [μm]	Density [g/cm^3]
A1.1	90	110	0.76
A1.2	120	174	0.64
A1.3	150	221	0.63
A2.1	90	101	0.83
A2.2	120	184	0.61
A2.3	150	230	0.61
A3.2	120	155	0.72
A3.3	150	208	0.67
A4.2	120	170	0.66
A4.3	150	223	0.63
B.1	150	630	0.22
B.2	200	874	0.21
B.3	250	1056	0.22
C.1	150	144	0.97
C.2	200	164	1.13
C.3	250	197	1.18
D.1	250	220	1.06
D.2	300	292	1.13
E.1	90	60	1.4
E.2	120	80	1.4
E.3	150	103	1.35
E.4	200	136	1.37
E.5	250	182	1.28
E.6	300	217	1.29
F.1	90	72	1.16
F.2	120	98	1.14
F.3	150	128	1.09

Table S.1: Table presenting the bulk calculations for the films in terms of thickness, weight and density.

S6 Calibration Methodology

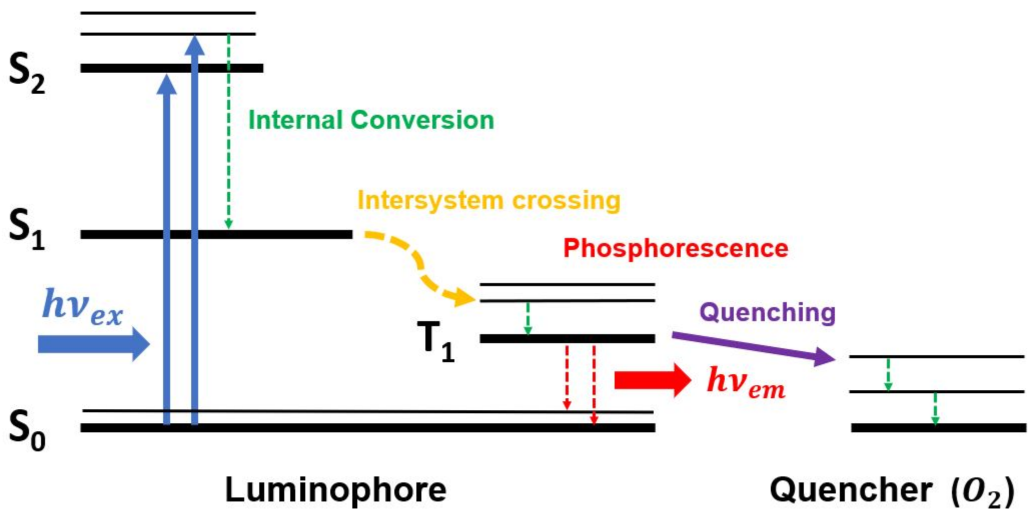

Fig. S6.1: Perrin-Jablonski diagram describing the quenching of phosphorescence from a luminophore by molecular oxygen.

The physical mechanism of oxygen sensing employed here is based on quenching of phosphorescence This process results in the different levels of light intensity reaching the photodiode depending on the oxygen concentration of the environment. This can be explained and visualised using the Perrin-Jablonski diagram (Fig. S6.1) [1]. In detail, Fig. S6.1 schematically depicts the energy states (S_0, S_1, S_2, T_1) and the different vibrational energy levels of each state vertically, while horizontally the different scenarios of de-excitation paths are represented for both the luminophore and the quencher. This type of schematic representation is described as a Jablonski diagram, where specific combinations of non-radiative and radiative relaxation paths are shown to describe luminescent phenomena.

Here, the luminescence phenomenon is phosphorescence ($h\nu_{em}$, red color), which occurs after the excitation ($h\nu_{ex}$, blue color) of the luminophore. Also, two different non-radiative relaxation processes are presented: internal conversion due to vibrational relaxation (spin allowed process) and intersystem crossing (spin forbidden process). In Fig. S6.1, the quenching of phosphorescence is depicted using oxygen (O_2 as the quencher) and the quenching non-radiative process is highlighted.

Phosphorescence quenching takes place when an intermolecular interaction between the luminophore and the quencher results in a non-radiative deactivation of the excited state of the luminophore and the excitation of the quencher's electrons. Such a process can be the result of collisional interactions or energy transfer. Both have been shown in the literature [1].

In essence, the after-effect of this intermolecular interaction is the non-radiative intersystem crossing of the luminophore to a lower energy triplet state T_1 before de-excitation to the ground energy state (radiative decay). This is accompanied by the production of a higher state oxygen (singlet state), a process that can be described by [2]:

$$L^* + O_2 = {O_2}^* + L + radiation \tag{S6.1}$$

where * represents the excited state, and L and O_2 are the luminophore and the oxygen molecule (which acts as a quencher), respectively.

This, in turn, results in phosphorescent light being emitted with a different intensity ([3]) and decay lifetime ([4]) depending on the presence and the amount of oxygen; best described in terms of concentration. This relation is usually presented in the form of the Stern-Volmer equation and is described in the main paper (see discussions around Eq. 1, section 1).

In this study, the oxygen sensing characterisation of the films was based on a standardised calibration procedure: 8 different oxygen concentrations (V/V) were tested in order to relate the light intensity received at the photodiode to the sensing performance. This involved the use of the mass flow controllers to control the oxygen concentration inside the enclosure at different levels (see main part of the paper section 2.3, Methodology). In Figs. S6.3 and S6.2a, the voltage measured in the oscilloscope from the photodiode is plotted over time for the entire process for a reflection based configuration for the film B.2. It should be underlined that the first oxygen plateau corresponded to 20% of oxygen (V/V) and the second to 0% (V/V) to deduce the

recovery time (see also section S1.d for definition). The plateaus correspond to constant oxygen concentrations of; 0, 5, 10, 20, 40, 60, 80 and 100% of oxygen flow (V/V).

Each point corresponding to a different oxygen concentration was then used to plot the Stern-Volmer graph (Figs. S6.2a and S6.2c). This was then fitted to Eq. 2 (section 1, described in the main part of the paper) using the open Python library Scipy (Figs. S6.2b and S6.2d). The R^2 values are shown in Figs. S6.2b and S6.2d as a measure of the fitting errors.

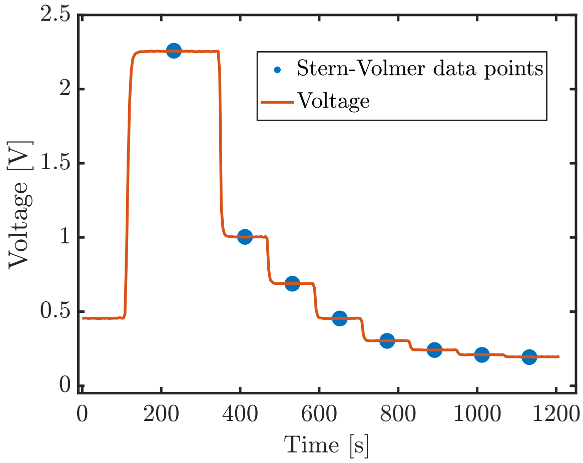

(a)

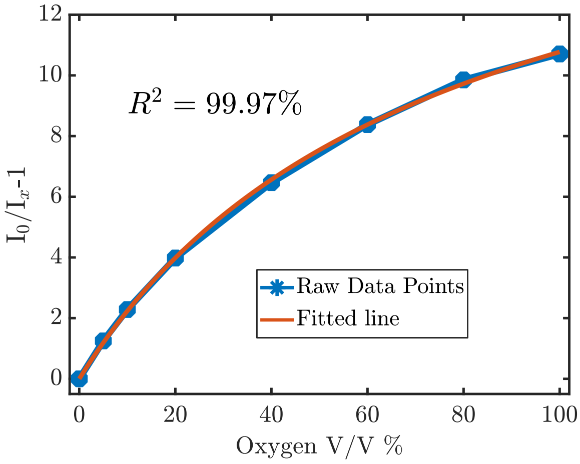

(b)

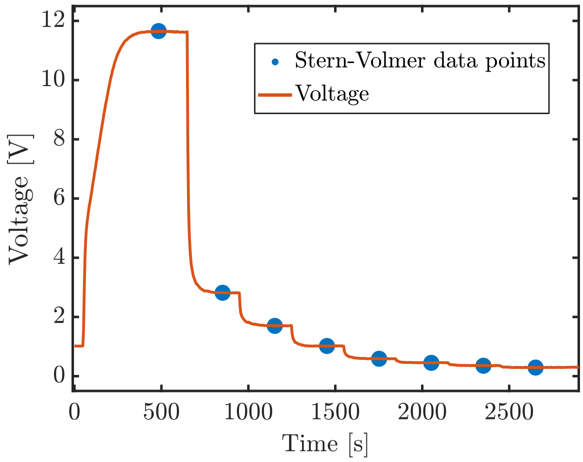

(c)

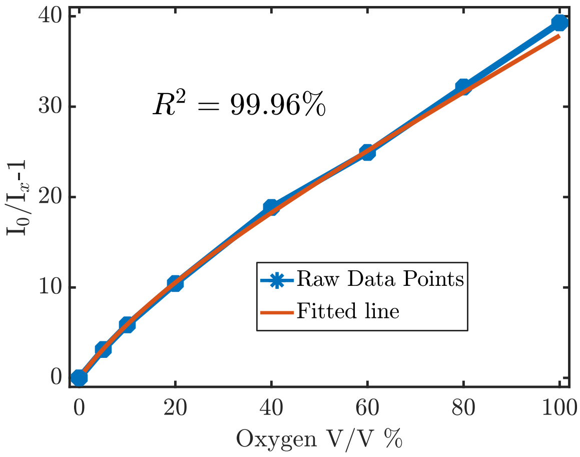

(d)

Fig. S6.2: a) Plot of the voltage output of the photodiode versus the time for the calibration experiments (red line), for film B.2 in the reflection-based configuration. The 8 data points corresponding to the different oxygen concentrations tested (0, 5, 10, 20, 40, 60, 80 and 100% V/V), and used for creating the Stern-Volmer plot are also indicated (blue points). b) Graph of the Stern-Volmer plot where $I_0/I_x - 1$ is plotted against the oxygen concentration V/V using the data points marked in (a). Both the raw data and the fitted line using the two site model (Eq. 2, main part of the paper) are displayed in blue and red, respectively. c) Plot of the voltage output of the photodiode versus the time for the calibration experiments (red line), for film D1 in the transmission-based configuration. The data points used for the Stern-Volmer plot are also indicated (blue points). d) Graph of the Stern-Volmer plot where $I_0/I_x - 1$ is plotted against the oxygen concentration V/V using the data points marked from (c). Both the raw data and the fitted line using the two site model (Eq. 2, main part of the paper) are displayed in blue and red, respectively.

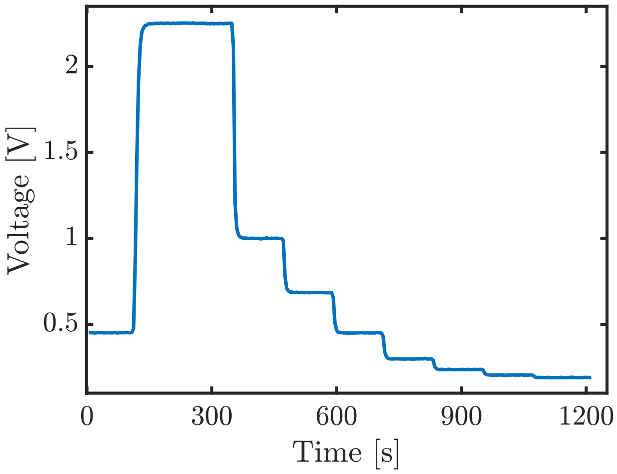

Fig. S6.3: The calibration test performed for film B.2 presented by showing the voltage over the time.

S7 Image Analysis: Digital Microscopy

Digital microscopy was the first imaging technique employed to depict the pore morphology of the films. In Fig. S7.1 two images of the film A2.2 are presented for the magnification lenses $\times$ 20 (a) and $\times$ 50 (b). For the calculation of the macrovoid dimensions, the image of the top surface of film A2.2 with a magnification lens of $\times 20$ (Fig. S7.2a) along with a blob detection technique were used (see section 2.3 Methodology, in the main part of the paper). This required the use of grayscaling, median filtering and contrasting of the image before the identification of the size and number of blobs. Each step of this process is presented in Fig. S7.2.

The results from the blob size calculations corresponding to the macrovoid sizes are presented in a histogram of the number of macrovoids detected over their diameter in Fig. S7.3.

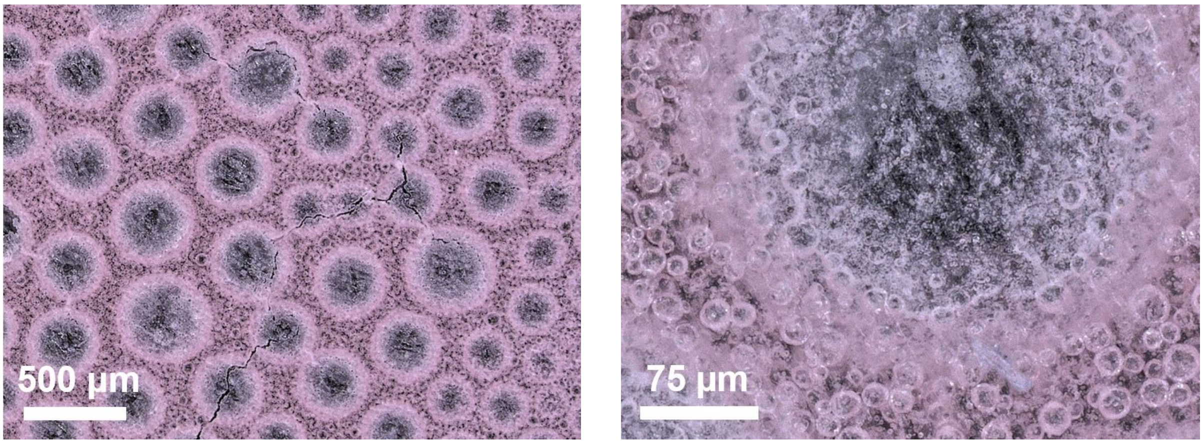

(a) (b)

Fig. S7.1: Images of the pores in film A2.2 obtained with digital microscopy for two different magnification lenses; a) $\times$20 and b) $\times$50.

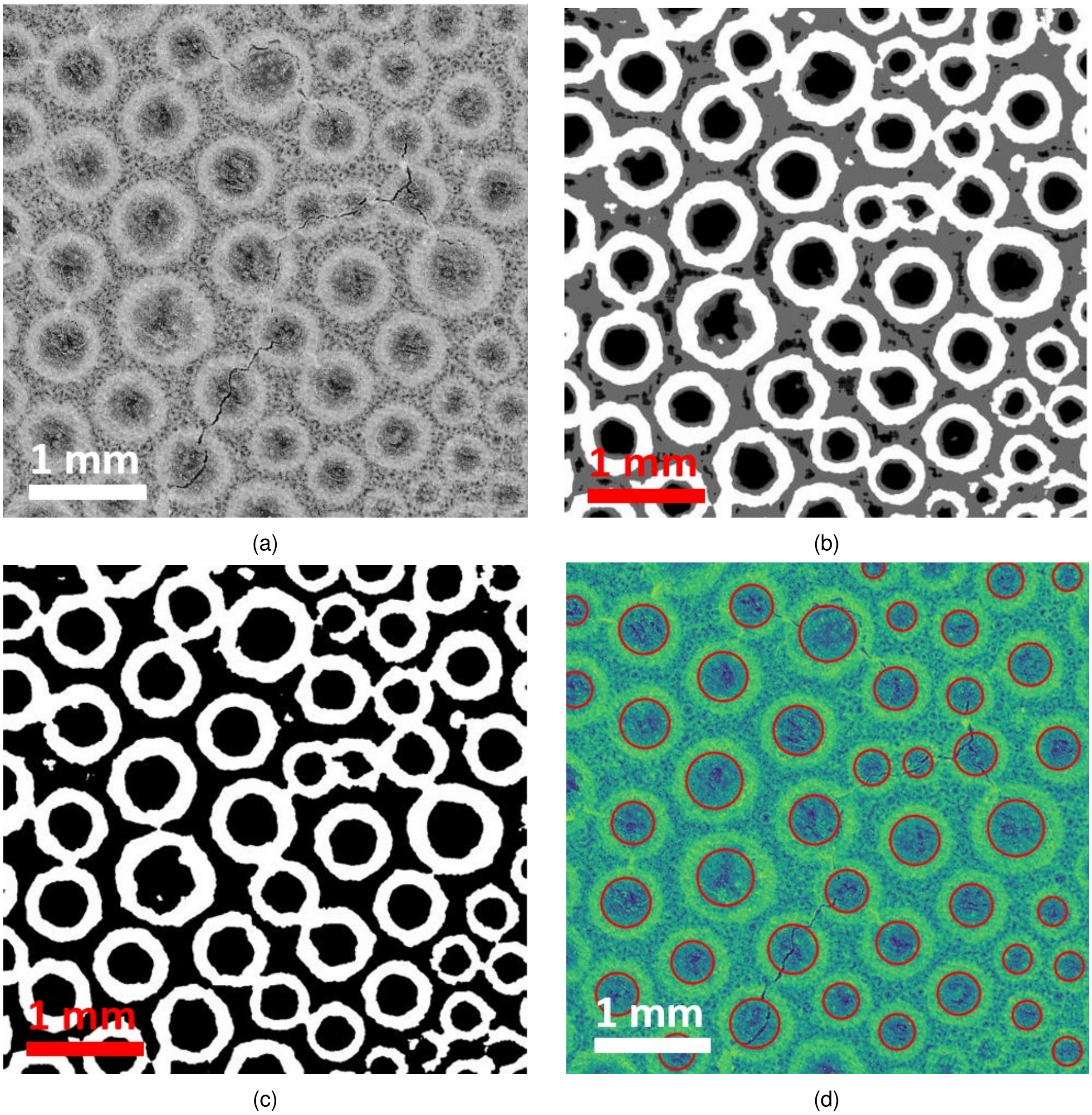

Fig. S7.2: Images of the pores in film A2.2 obtained with digital microscopy for the lens ×20 after the application of a) grayscaling b) contrast enhancement and filtering c) thresholding and d) blob identification (red circles).

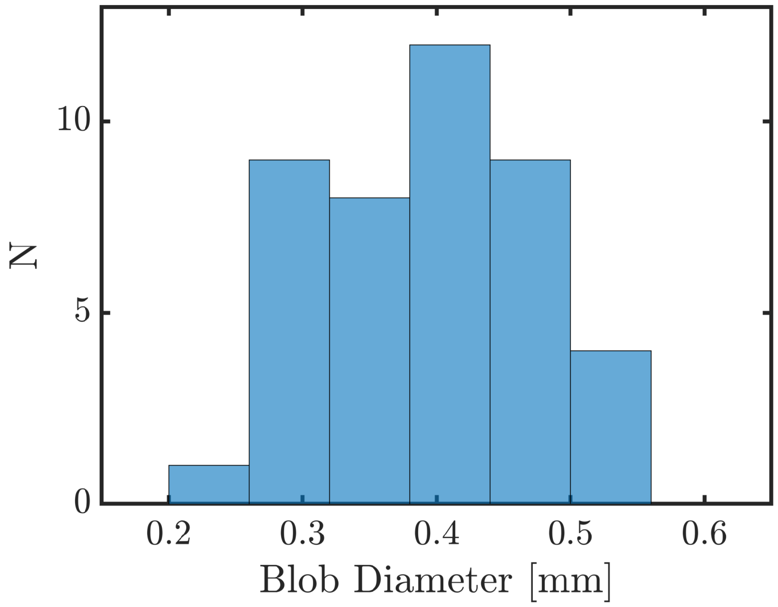

Fig. S7.3: Histogram of the blob diameter detected in the image depicting the pores in A2.2 obtained with digital microscopy for the magnification lens ×20.

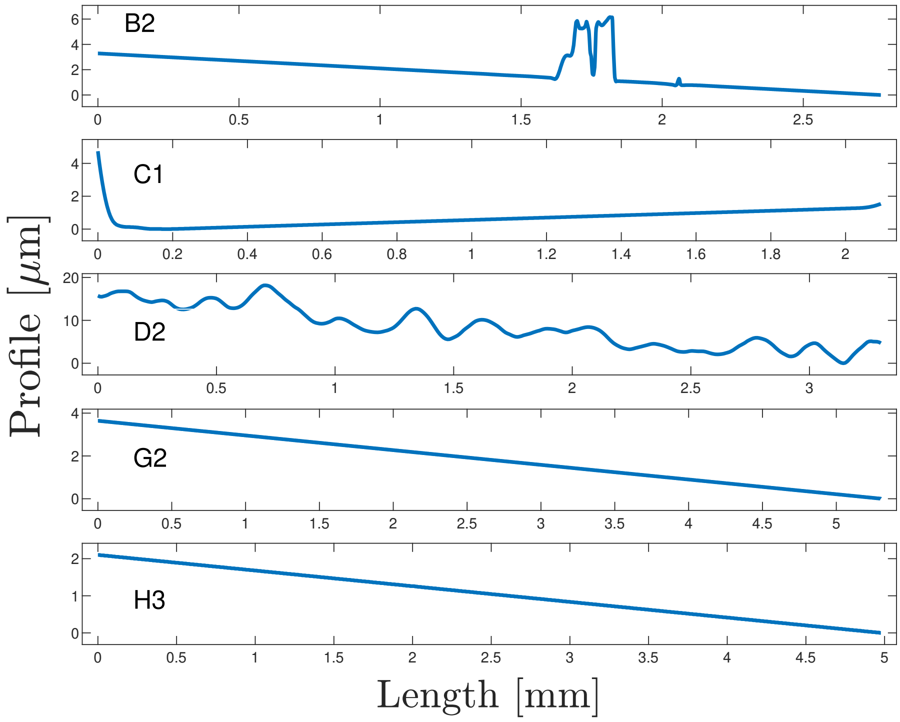

Fig. S7.4: Plot of the profile of the surface of films B2, C1, D2, G2 and H3 over the length of measurement. Each plot is annotated with the respective film type. These calculations were deduced from the 3D representation of the films.

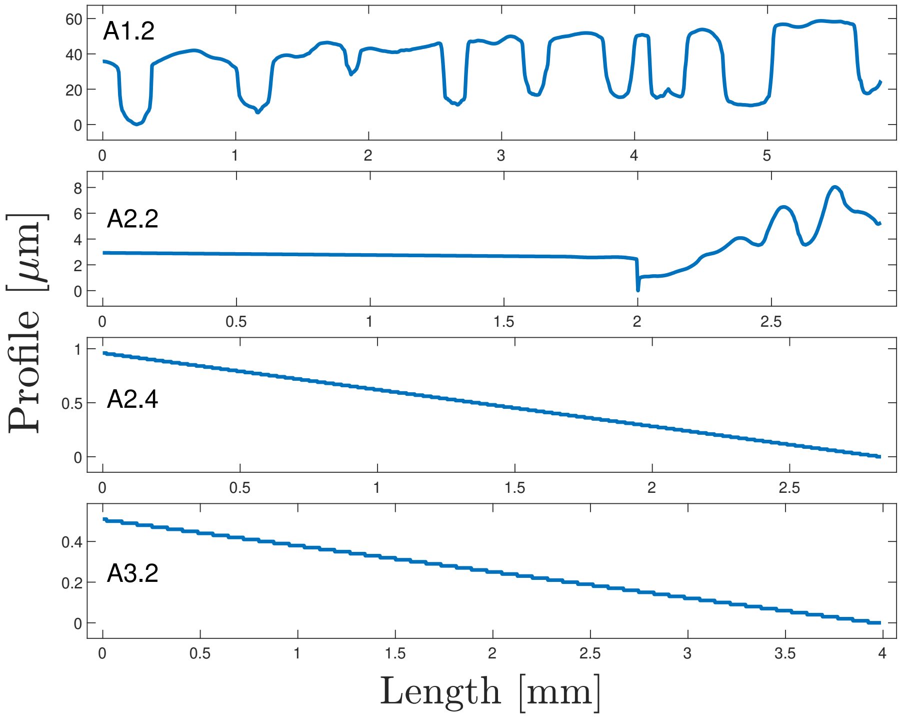

Fig. S7.5: Plot of the profile of the surface of films A1.2, A2.2, A2.4 and A3.2 over the length of measurement. Each plot is annotated with the respective film type. These calculations were deduced from the 3D representation of the films.

Additionally, 3D representations of the films were obtained using digital optical microscopy and the profiles of the films in the z direction were calculated (shown in Figs. S7.4 and S7.5). The results were plotted as profile versus the length of the film that the measurements were collected in the x-y plane (Fig. S2.2). Figs. S7.4 and S7.5 clearly show that the thickness changes are small (up to 4μm) for films A1.2, A2.2, A2.4, A3.2, B2, C1, G2 and H3. For film A1.2, the measurements were taken over open pores and this resulted in plateaus and valleys. This was performed in order to show that the macrovoids had an impact on the surface roughness. Nonetheless, the change in thickness on the top of the plateaus is similar to the other films in the order of 10 μm. For film D2 (Fig. S7.4) the variations in profile were up to 20 μm, which indicated increased roughness and possible curving of the D film during the drying procedure. In all cases, these results show that the coffee ring effect can be ignored since the changes in thickness are considered negligible.

S8 Image Analysis: micro-CT

The second imaging technique used for the morphological characterisation of the films was micro-CT (see also section 3.1, Morphology Characterisation - Image analysis). The raw images (with the addition of their scalebars) obtained with this technique are depicted in Fig. S8.1, for film A2.2 and Fig. S8.2 for B.2.

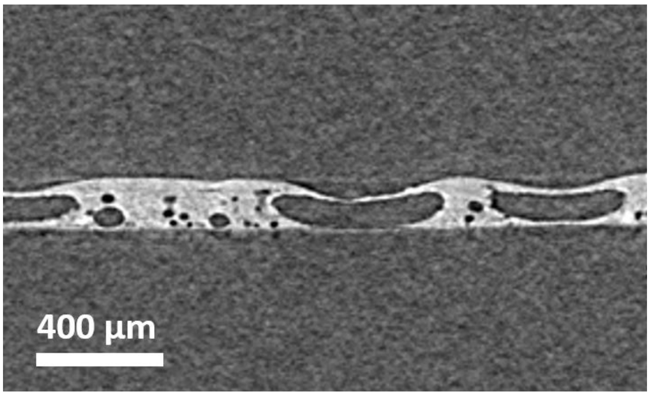

Fig. S8.1: Image of the side-view of film A2.2 obtained with micro-CT.

For film A2.2, the two blobs identified had a diameter of ~ 354 µm and ~ 460 µm, which was within the range of observed values seen in the images obtained with DM (Fig. S7.3). Additionally, the two large blobs (as also seen from DM shown in Fig. S7.1b) were identified to go through the entire z direction leaving only thin connections with the bottom and top surface. It is noteworthy that SEM images of the top surface of the film indicated that a number of them were open (not shown here for brevity).

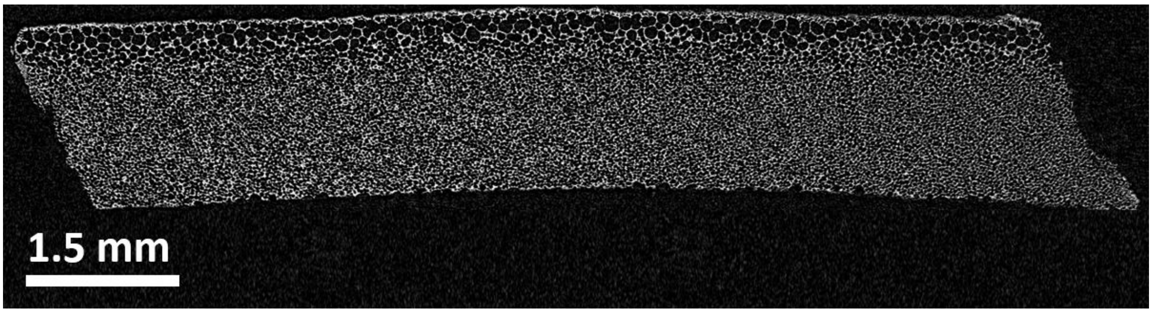

Fig. S8.2: Raw image of film B.2 obtained with micro-CT for a magnification a magnification of 555%.

Once more, grayscaling and thresholding as well as contrasting (images before and after processing are shown in Fig. 4 of the main part) was required to determine the porosity and the pore sizes of the films. For film B.2, the threshold chosen was determined using Eqs. S8.1 and S8.2 (Isodata threshold) :

$$m(t) = \frac{M_L + M_H}{2} \tag{S8.1}$$

where t is the threshold, M_L is the mean of all pixels in the image with a gray value less then or equal to t and M_H greater than t.

$$t_{i+1} = m(t) \tag{S8.2}$$

where i is the value of an iteration that goes through all the points in the histogram until the threshold is the closest value to m(t).

Also, the definition of the porosity as a function of black and white pixels is presented in Eq. S8.3. It should be noted that this calculation was performed after the contrasting and grayscaling of the micro-CT images.

$$Porosity = \frac{N_B}{N_B + N_W} \times 100\% \tag{S8.3}$$

where N_B is the number of black pixels and N_W of white pixels.

The successful characterisation of film B.2 in terms of porosity and distribution of average Feret diameter is described in the main part of the paper (Fig. 4, section 3.1, Morphology Characterisation - Image analysis).

Here, the histogram depicted in Fig. S8.4 presents the number of Feret diameters measured versus their size. There is a clear trend of diminishing number of Feret diameters with increasing size. Also, there exists a peak in the range of 30-35 μm, while the minimum diameter resolution was 10 μm and the maximum diameter range 140-145 μm.

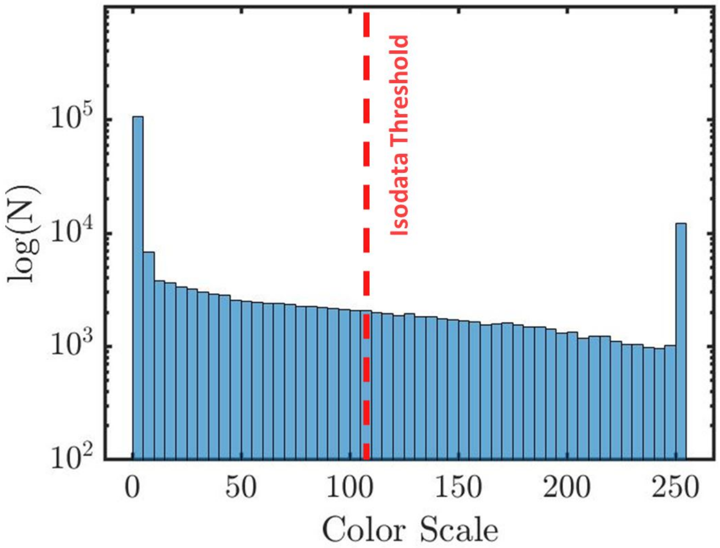

Fig. S8.3: Plot of the histogram of the color scale for the micro-CT image of the B film with the threshold indicated with a red dashed line

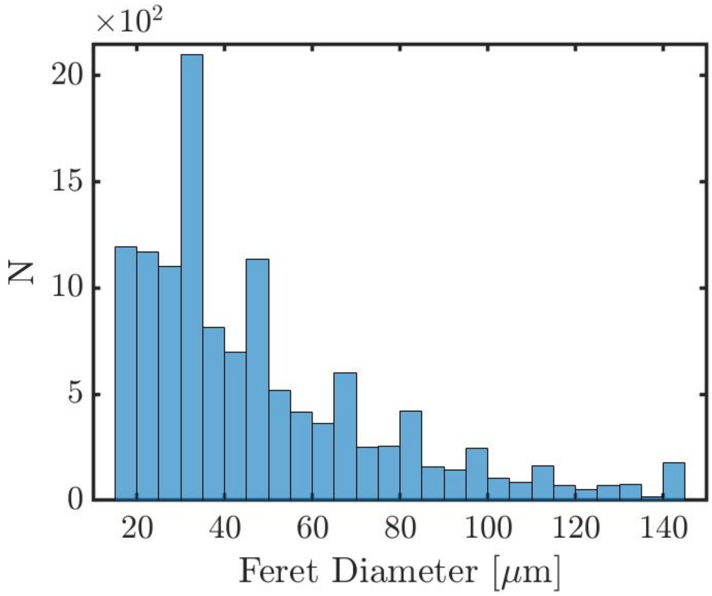

Fig. S8.4: Histogram of the length of the Feret diameter above the minimum diameter resolution ($\sim$10μm for the micro-CT image of the B film).

S9 Oxygen Sensing

As mentioned in the main part of the paper, the oxygen sensing characterisation was based on: 1) maximum sensitivity, 2) recovery time, 3) half-sensitivity point and 4) dynamic range (see also section S1 for definitions). These properties are presented in tables S.2 and S.3 for porous and dense films, respectively, for a reflection based configuration. The weight, thickness and density of the films are also shown.

It should be noted that the low recovery of B.1 (8s) is possibly a result of experimental error (random or systematic since the resolution was low, i.e. ± 4s) because the stabilisation of the concentration in the enclosure through mass flow control was achieved after $\sim$ 10 s, as verified with the use of the amperometric reference device. Another explanation could be that the response of this film was quicker than the reference oxygen sensing method. Also, despite the longer recovery times, the D films showed improved sensing properties compared to the ternary based fabricated films in terms of DR and half-sensitivity points, but the amount of polymer and thus the number of dye molecules was higher (300 mg versus 250 mg for B.3). It should be further noted that E.2 did not follow the trend of increasing sensing performance with increasing amount of polymer possibly due to experimental error during the fabrication procedure, which is also supported from the lower recovery time it exhibited (table S.2). Relatively small differences in density in E.5 and E.6 films can be explained by the decreased coffee ring effect for higher polymer concentrations, but this was not quantitatively determined and is out of the scope of this study.

Moreover, the dense films performed worse compared to their porous counterparts for all sensing properties analysed, apart from the recovery time which remained to a large degree unaltered. Specifically, upon inspection, their $O_2(S = S_{max}/2)$, DR and S_{100} were much lower when compared to the porous films with the equivalent amount of polymer. In addition, the increase in polymer amount resulted in increase in the S_{100}, DR, $O_2(S = S_{max}/2)$ and recovery times, as expected, but in all cases this was not significant enough to surpass the properties of the porous films.

It is noteworthy that although dense polystyrene based films showed relatively high sensitivity for 120 mg of polymer, they also presented the highest recovery times and a low DR. Moreover, the density of CAB (type E) and PS (type F) dense films was similar in all cases of polymer amount and were higher compared to the porous films (A, B, C, D), as expected, due to the lack of porosity.

Film	Polymer [mg]	Thickness [µm]	Density [g/cm^3]	I_0/I_{100}	$t\downarrow_{90\%}$ [s]	$O_2(S = S_{max}/2)$ [%]	DR [V]
A1.1	90	110	0.76	4.79	64	4	0.37
A1.2	120	174	0.64	6.93	124	8	0.62
A1.3	150	221	0.63	8.15	236	10	0.85
A2.1	90	101	0.83	6.17	44	7	0.52
A2.2	120	184	0.61	7.53	104	8	0.68
A2.3	150	230	0.61	8.11	156	9	0.85
A3.2	120	155	0.72	5.10	60	5	0.38
A3.3	150	208	0.67	7.80	172	8	0.72
A4.2	120	170	0.66	7.00	144	7	0.68
A4.3	150	223	0.63	9.16	264	10	0.90
B.1	150	630	0.22	11.66	8	26	1.90
B.2	200	874	0.21	11.89	24	26	2.06
B.3	250	1056	0.22	12.00	24	27	1.68
C.1	150	144	0.97	9.19	56	10	1.01
C.2	200	164	1.13	9.17	156	10	1.05
C.3	250	197	1.18	10.24	276	12	1.23
D.1	250	220	1.06	11.20	188	13	0.96
D.2	300	292	1.13	12.43	292	15	1.19

Table S.2: Oxygen sensing properties of the porous films for a reflection based configuration.

A comparison between porous and dense films was performed to calculate the improvement in the sensing properties (Eqs. S9.1 - S9.4). The definitions proposed for this purpose were: the relative change in a) the maximum sensitivity ($\Delta S_{100}/S_{100}^d$, Eq. S9.1) b) the recovery time ($\Delta t\downarrow_{90}/t\downarrow_{90}^d$, Eq. S9.2) and c) dynamic range ($\Delta(DR)/DR_d$, Eq. S9.3) and d) the absolute change in $O_2(S = S_{max}/2)$ ($\Delta O_2(S = S_{max}/2)$, Eq. S9.4). The results from these calculations are shown in tables 2 and 4, presented in the main part of the paper.

$$\Delta S_{100}/S_{100}^d = \frac{S_{100}^p - S_{100}^d}{S_{100}^d} \quad \text{(S9.1)}$$

$$\Delta t\downarrow_{90}/t\downarrow_{90}^d = \frac{t\downarrow_{90}^d - t\downarrow_{90}^p}{t\downarrow_{90}^d} \quad \text{(S9.2)}$$

Film	Polymer [mg]	Thickness [μm]	Density [g/cm^3]	I_0/I_{100}	$t\downarrow_{90\%}$ [s]	$O_2(S = S_{max}/2)$ [%]	DR [V]
E.1	90	60	1.40	4.02	36	2	0.26
E.2	120	80	1.40	3.58	28	2	0.26
E.3	150	103	1.35	4.19	52	2	0.29
E.4	200	136	1.37	4.94	88	3	0.38
E.5	250	182	1.28	6.48	208	5	0.53
E.6	300	217	1.29	7.00	$\sim$ 286	6	0.60
F.1	90	72	1.16	3.83	164	6	0.44
F.2	120	98	1.14	5.20	296	11	0.26
F.3	150	128	1.09	-	$<$ 400	-	-

Table S.3: Oxygen sensing properties of the dense films for a reflection based configuration.

$$\Delta(DR)/DR_d = \frac{DR_p - DR_d}{DR_d} \tag{S9.3}$$

$$\Delta O_2(S = S_{max}/2) = O_2(S = S_{max}/2)^p - O_2(S = S_{max}/2)^d \tag{S9.4}$$

where the notations d, p represent the dense and porous films respectively.

Notably, due to experimental error during the fabrication procedure the thicknesses and thus the density were slightly different when comparing the films produced for transmission and reflection based configurations (tables S.2 and 3 in the main part of the paper). As stated in the main part of the paper (section 2.3), the two sets of experiments used films that were fabricated separately to avoid the effects of ageing and photobleaching. Specifically, from tables S2, S3 and 3 (main part) it is clear that this led to small differences in the recovery times of films which were produced with the same fabrication procedures on separate occasions.

S10 Humidity Sensitivity

The variations in performance due to different humidity levels were tested in terms of voltage difference induced by monitoring a single oxygen concentration in high (V_h) and low (V_{21}) humidity environments (ΔV_h, Eq. S10.1). Additionally, in order to estimate the effect on the oxygen sensing capabilities at that specific oxygen concentration; ΔV_h is compared with the derivative of the calibration curve which relates the voltage at the oscilloscope to the oxygen concentration, after fitting with the two site model. The derivative is chosen at the specific oxygen point where the experiments were performed (i.e 21%) and corresponds to the predicted difference in voltage measurements for a 1% incremental change in oxygen flow. For this type of calculations, a linear relationship is assumed between two adjacent points in the calibration curve. Thus, by exploiting the derivative at that point the error in the oxygen measurement is given as the ratio of the induced difference in voltage due to humidity variation over the predicted change for a 1% variation in percentile flow (ΔO_2%, Eq. S10.2). In essence, this corresponds to a error based on the predicted difference in voltage measurements for a 1% incremental change in oxygen flow over the experimentally measured voltage difference induced by humidity. Also for these calculations to be valid a linear relationship is assumed between two adjacent points in the calibration curve. The measurements were performed with a reflection based setup. The results for these calculations are presented in Table S.4 for films A2.2, B2, C.2, D.1, E.2 and F.2.

$$\Delta V_h = V_h - V_{21} \tag{S10.1}$$

where V_h is the voltage at the oscilloscope during the high humidity experiment and V_{21} is the voltage when the oxygen concentration was set to 21% V/V (low humidity environment due to the use of the mass flow controllers and the gas tanks).

$$\Delta O_2\% = \frac{\Delta V_h}{\left(\frac{dV}{d_{O_2}}\right)_{21}} \times 1\% \tag{S10.2}$$

where $\left(\frac{dV}{d_{O_2}}\right)_{21}$ is the derivative of the calibration curve which relates the voltage at the oscilloscope to the oxygen concentration at 21% V/V, after fitting with the two site model.

Film	ΔV_h [mV]	$\Delta V_h/DR[\%]$	DV_{21} [mV]	ΔO_2%
A2.2	2.0	0.29	2.3	0.87
B.2	4.7	0.23	15.7	0.30
C.2	1.9	1.80	4.8	0.40
D.1	1.7	1.77	6.2	0.27
E.2	0.7	2.69	1.1	0.64
F.2	0.5	1.92	4.5	0.11

Table S.4: Relative humidity influence on the oxygen sensing properties of the films (6 indicative films were chosen).

S11 Surface Area calculations from Bulk changes

By relating the changes in volume and the average pore diameter (eq. S11.1 and S11.2) it is possible to get an estimation of the change in surface area between dense and porous films (eq. S11.4):

$$|\Delta V| = (h_B - h_f) \times A, \quad A = \frac{\pi \times D_{film}^2}{4} \tag{S11.1}$$

where ΔV is the change in volume, h_B and h_F are the heights of B.1 and F.3 and A, D_{film} the area and diameter of the films.

Thus, by assuming that the change in volume is only a result of the pore formation, that the pores have a spherical shape and by using the average pore diameter from micro-CT calculations, we can find the number of the pores:

$$|\Delta V| = N \times \frac{\pi D_{av}^3}{6} \tag{S11.2}$$

And by assuming that the surface area change is only due to the pores:

$$\frac{|\Delta(SA)|}{SA} = \frac{N\pi D_{av}^2}{\pi D_f h_f + \frac{\pi}{2} D_f^2} \tag{S11.3}$$

Thus combining equations:

$$\frac{|\Delta(SA)|}{SA} = \frac{3D_f}{2D_{av}} \frac{h_B - h_F}{h_f + D_f/2} \cong \frac{3(h_B - h_f)}{D_{av}} \tag{S11.4}$$

Thus for films B.1 and F.3 (with the assumption of the same average pore size of B.1 and B.2) the value is ~ 31.3.

S12 Reflectance and Transmittance tests

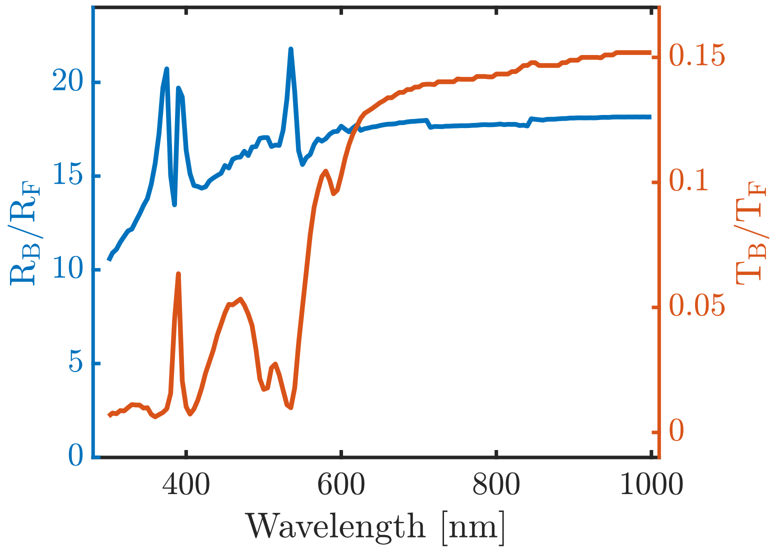

Fig. S12.1: Plot of the reflectance (blue) and transmittance (red) ratios of film B.1 over F.3 versus the wavelength of the excitation light.

The most probable reason behind the differences in sensing properties from one configuration to another (reflection vs transmission) was the variation of transmittance and reflectance properties of the films under study. To demonstrate this, additional experiments were performed with the dense F.3 film and the B.1 film, which had the same amount of polymer in terms of weight (150 mg). In detail, using UV–visible spectrophotometry, the total transmitted and reflected light was measured and compared for both types of films, using equal amount of polymer/dye concentration (W/W). The comparison was performed by looking at the ratios of films B.1 and F.3 in terms of transmittance (T_B/T_H) and reflectance (R_B/R_H), over the range of excitation light from 300 to 1000 nm (Fig. S12.1). It should be noted that this was performed to compare between the dense and porous film proposed in this study and these measurements do not reflect the properties of the films without the dyes.

From Fig. S12.1 it is clear that the reflected light from the B film is much larger compared to its dense counterpart for the entire spectrum tested (maximum of ~ 21). In contrast, the equivalent ratio for the transmitted light is significantly smaller than 1 (minimum of $\sim$ 0.01). This is a clear indication that B films would be ideal for reflection based measurements but not for transmission. Additionally, sharp peaks at the absorption wavelengths of the dye molecules (~ 390 and ~ 530 nm) were observed for the reflection ratio, and valleys at the equivalent wavelengths for the transmission ratio. Thus, apart from the increased reflectance or transmittance of one film versus the other, the sharp rise and fall in the light reaching the detector (at specific wavelengths) indicates that additional differences might be present due to the absorption and emission from the dye molecules in each film. To verify this, additional studies are required by including tests without embedding the dyes into the films.

There are strong suggestions from the literature that differences between the transmittance and reflectance properties was a result of the pore formation [5]. It is assumed also that the asymmetrical nature of the films produced with both methods [6, 7, 8] and their differences in terms of thickness contributed to this effect.

References

[1] Michela Quaranta, Sergey M Borisov, and Ingo Klimant. Indicators for optical oxygen sensors. *Bioanalytical reviews*, 4(2-4):115–157, 2012.

[2] J NI Demas, BA DeGraff, and Wenying Xu. Modeling of luminescence quenching-based sensors: comparison of multisite and nonlinear gas solubility models. *Analytical Chemistry*, 67(8):1377–1380, 1995.

[3] John I Peterson, Raphael V Fitzgerald, and Delwin K Buckhold. Fiber-optic probe for in vivo measurement of oxygen partial pressure. *Analytical chemistry*, 56(1):62–67, 1984.

[4] Jane M Vanderkooi and David F Wilson. A new method for measuring oxygen concentration in biological systems. In *Oxygen transport to tissue VIII*, pages 189–193. Springer, 1986.

[5] Jyotirmoy Mandal, Yanke Fu, Adam C Overvig, Mingxin Jia, Kerui Sun, Norman N Shi, Hua Zhou, Xianghui Xiao, Nanfang Yu, and Yuan Yang. Hierarchically porous polymer coatings for highly efficient passive daytime radiative cooling. *Science*, 362(6412):315–319, 2018.

[6] Klaus-Viktor Peinemann, Volker Abetz, and Peter FW Simon. Asymmetric superstructure formed in a block copolymer via phase separation. *Nature materials*, 6(12):992–996, 2007.

[7] Sacide Alsoy Altinkaya and Bulent Ozbas. Modeling of asymmetric membrane formation by dry-casting method. *Journal of Membrane Science*, 230(1-2):71–89, 2004.

[8] Bülent Özbaş. *Modelling of asymmetric membrane formation by dry casting method*. Izmir Institute of Technology (Turkey), 2001.
